# TRAIL (DR5) receptor and the modulation of TRAIL pathway in PLWHIV: key mechanisms in the progression of HIV disease

**DOI:** 10.1186/s12860-025-00541-z

**Published:** 2025-06-01

**Authors:** Sarah Ratkovich-Gonzalez, Mariana Del Rocio Ruiz-Briseño, Judith Carolina De Arcos-Jiménez, Monserrat Alvarez-Zavala, Jaime Federico Andrade-Villanueva, Pedro Martínez-Ayala, Vida V. Ruíz-Herrera, Luz Alicia Gonzalez-Hernandez, Karina Sánchez-Reyes

**Affiliations:** 1 Monterrey Institute of Technology and Higher Education, School of Medicine and Health Sciences, Avenida General Ramón Corona 2514 Nuevo México, Zapopan, Jalisco 45138 Mexico; 2 Department of Technological and Industrial Processes, Western Institute of Technology and Higher Studies (ITESO), Anillo Perif. Sur Manuel Gómez Morín 8585, Santa María Tequepexpan, San Pedro Tlaquepaque, Jalisco 45604 Mexico; 3 Department of Health-Disease as an Individual and Collective Process, CUTLAJO, University of Guadalajara, Carretera Tlajomulco, Santa Fé Km. 3.5 No.595, Lomas de Tejeda, Tlajomulco de Zúñiga, Jalisco C. P. 45641 Mexico; 4 State Laboratory of Public Health, Jalisco Health Secretary, Av. Zóquipan No.- 1000, Edificio “B”, Colonia Seattle, Zapopan, Jalisco C.P. 45170 Mexico; 5 HIV and Immunodeficiencies Research Institute (InIVIH), Department of Medical Clinics, CUCS, University of Guadalajara, Hospital 278, Guadalajara, Jalisco CP 44280 Mexico; 6 HIV Unit Department, Antiguo Hospital Civil de Guadalajara “Fray Antonio Alcalde”, Hospital 278, Guadalajara, Jalisco CP 44280 Mexico

**Keywords:** HIV, Fas, FasL, TRAIL, DR5, IL-18, Inflammation, Apoptosis, Co-receptors

## Abstract

**Background:**

HIV infection is mainly described by depletion of CD4^+^ T-cells; however, this not only occurs in infected cells, also arise in uninfected immunological cells through the bystander effect. Extrinsic cell death, in particular the Fas pathway has been studied in HIV extensively, and an expression increase in both its ligand and receptor has been reported, however the TRAIL pathway has been less explored in this context, and little has been relating to the immune activation characteristic of the disease. This study aims to examine the effect of HIV infection in the activation of TRAIL and Fas death pathways in CD3^+^ CD4^+^ T-cells and CD4^+^ CD14 + monocyte derived from people living with HIV (PLWHIV) and its correlation with immune activation biomarkers in cell surface and serum.

**Results:**

Expression of TRAIL receptor DR5 in CD3^+^ CD4^+^ T-cells and CD14^+^ CD4^+^ monocytes from PLWHIV were significatively increased, almost two and five times more than CD3^+^ CD4^+^ T-cells and CD14^+^ CD4^+^ monocytes from HIV-negative controls; respectively. In PLWHIV, DR5 and CCR5 expression were positively and negatively associated with time of infection; respectively. Simultaneously, DR5 was associated positively with CXCR4 expression in CD3^+^ CD4^+^-T cells and CD4^+^ CD14^+^ monocytes as well as the significant increase of serum levels of IL-18 in PLWHIV. In CD3^+^ CD4^+^-T cells from HIV patients, the expression of CD38 was upregulated. Finally, in CD14^+^ CD4^+^ monocytes from PLWHIV, it was observed an increase in early apoptosis in response to recombinant TRAIL ligand, an effect that was not inhibited by caspase 8 blockade.

**Conclusions:**

In PLWHIV before ART, the activation and regulation of TRAIL pathway shows to be an important regulator in cell depletion. The expression of TRAIL DR5 significantly increased in CD3^+^ CD4^+^-T cells and CD4^+^ CD14^+^ monocytes from PLWHIV; in the same way DR5 was positively correlated with time of infection, with CXCR4 expression and with the significant increase in serum levels of IL-18, making it an interesting target for future treatments and as a marker for HIV disease progression.

## Background

The HIV infection induces a progressive depletion of the CD4^+^ T-cells, this decrease leads to immunodeficiency, a condition that increases the risk of opportunistic infections and malignancies [1]. Depletion cell occurs through various mechanisms in both, infected and uninfected CD4^+^ T-cells, as well as other immune cells such as immature thymocytes, CD8^+^ T-cells, B cells, monocytes among others [2, 3]. One of the main mechanisms of the cell death induced by HIV infection is the cytopathic effect and bystander effect; however, other mechanisms has been proposed, like those that involve extrinsic and intrinsic pathways, particularly apoptosis and pyroptosis [4]. Apoptosis is a mechanism of programmed cell death, that is classified according to the nature of the triggering stimulus, in intrinsic or extrinsic pathway; the latter can be induced by a death ligands as tumor necrosis factor (TNF), Fas ligand (FasL), or TNF-related apoptosis inducing ligand (TRAIL), engaging with their specific death receptor [5]. The HIV infection is characterized by a chronic immune activation and hyperactivation, which increase death ligand expression, favoring CD4^+^ T-cells depletion The “hyper immune activation” hypothesis in HIV infection suggests that, there is a high rate activation and cell proliferation of CD4^+^ and CD8^+^ T-cells, NK cells, and B cells, which is associated with the upregulation of activation markers; consequently, these cells have a decreased life period and become depleted quickly due to activation induced cell death or apoptosis [1, 6]. It has been described that immune activation, perpetuates over time even with a successful Antiretroviral Therapy (ART) that achieves suppression of HIV replication. This phenomenon is considered a hallmark of the HIV infection and is associated with the extensive apoptosis of T cells in response to viral products as Nef, Tat, Vpr, and Vpu, and the production of inflammatory cytokines in response to viral products and the presence of cytoplasmatic HIV DNA [1, 7].

Moreover, the high production of inflammatory cytokines such as type I interferons (IFNs), interleukin-1α (IL-1α), IL-1β, IL-6, IL-8, transformant growth factor-β (TGF-β), monocyte-1α (MIP-1α), MIP-1β, and RANTES causes the immune activation and eventually, the loss of CD4^+^ T-cells [1]. Particularly, it has been reported that IL-6 promotes T cell survival by increasing Bcl-2 expression; likewise, abortive infection induces T cell death by pyroptosis in the Gut-Associated Lymphoid Tissue (GALT), which releases IL-1β. High levels of cytokines favor the expression of death receptors. In this sense, high levels of the type I IFN results in elevated expression of TRAIL receptors and proapoptotic mediators and IL-18 promotes the Fas receptor (FasR) expression, and in turn the binding of Fas-FasL increase IL-18 levels [8].

In HIV infection, Fas pathway is considered the most important cell death mechanism of CD4^+^ T-cells depletion. Specifically, diverse immune cells, as CD4^+^ and CD8^+^ T-cells, as well as B-cells showed increased Fas receptor expression; also, the FasL expression is augmented in macrophages, NK cells, and monocytes, resulting in selective killing of uninfected CD4^+^ T-cells; this phenomenon has not been observed in non-progressing patients [9, 10]. In vitro assays, have demonstrated that HIV infected cells are more susceptible to Fas-mediated apoptosis; also, this pathway has been implicated in the “bystander effect” of uninfected cells [11, 12]. TRAIL, a TNF superfamily member, has apoptotic effects in tumorous and virus infected cells. There are five receptors to TRAIL, two of which (TRAIL-R1 or DR4 and TRAIL-R2 or DR5) are capable of inducing apoptosis after the formation of Death-Inducing Signaling Complex (DISC), and the remaining TRAIL-R3 or DcR1, TRAIL-R4 or DcR1, and the soluble receptor called osteoprotegerin (OPG) are considered anti-apoptotic and are called Decoy Receptors (DcRs) [13]. In the HIV immunopathogenesis, an increase in TRAIL expression in cells, especially in viremic patients, has been described [14, 15]; on the other hand, murine and in vitro studies have shown that this molecule can induce the death of uninfected and resting memory CD4^+^ T-cells, macrophages [16] as well as memory B-cells [17]. Moreover, it has been demonstrated that macrophages exposed to the HIV-1 transactivating (Tat) protein are producers of TRAIL, which in turn is capable of inducing apoptosis in uninfected CD4^+^ T-cells [12]. In relation to TRAIL receptors, it has been described that there is a high expression of DR5 in CD4^+^ T-cells of people living with HIV (PLWHIV) and that its blockade using anti-DR5 antibodies reduces apoptosis of these cells [18]. Also, it has been described that HIV infection reduces the TRAIL decoy receptors, thus, inducing apoptosis in macrophages [19]. On the other hand, a study performed with HIV-1 controllers (HIC) or elite controllers, a subset of PLWHIV who does not progress to AIDS and spontaneously maintains an undetectable viral load, showed that the CD4 + T-cells of HIC are deficient in DR5 cell surface expression after HIV-1 stimulation and they do not carry out apoptosis [19].

The objective of this study was to analyze in PLWHIV the effect of HIV infection, on the serum concentration of inflammatory cytokines, as well as effect of the virus on the expression of receptors and ligands of the Fas and TRAIL pathways, the expression of the coreceptors CXCR4, CCR5 and activation markers in CD3^+^ CD4^+^ T-cells and CD14^+^ CD4^+^ monocytes derived from PLWHIV before ART.

## Methods

### Subjects

Fourteen PLWHIV before ART, were recruited from the HIV Unit Department in Guadalajara’s Civil Hospital “Fray Antonio Alcalde”, Guadalajara, Mexico from March 2018 to April 2019. The inclusion criteria were: male, over 18 years, waist circumference < 90 cm, ART *naïve* and CD4^+^ T-cell count > 350 cells/µL. Also, the subjects that presents other viral infections, background of hematopoietic diseases, neoplastic diseases, autoimmune diseases, or the use of intravenous drugs were excluded. Further, fourteen non-HIV and clinically healthy volunteers, age and sex matched were recruited.

The investigation was approved by the Ethics Committee from Civil Hospital of Guadalajara “Fray Antonio Alcalde” and University of Guadalajara (approval number: 127/17). The CD4^+^ and CD8^+^ T-cells count, and the HIV-1 RNA viral load were determined by the State Reference Laboratory. Clinical data were obtained from the database of the HIV Unit Department in Civil Hospital of Guadalajara “Fray Antonio Alcalde”.

### Sample collection

With a previous informed consent, peripheral blood samples were obtained by venipuncture in tubes with K_2_EDTA as anticoagulant as well as in tubes with clot activator. After 10 min at room temperature, one tube with clot activator was centrifuged at 1800 *g* for 10 min. The serum obtained were aliquoted and stored at -80 °C until use or was used to determine the biochemical profile.

### Biochemical profile

To determine if the HIV-controls were clinically healthy, routine laboratory studies were performed in the Central Laboratory of Civil Hospital of Guadalajara “Fray Antonio Alcalde”. Serum lipids, including total cholesterol (TC), high-density lipoprotein cholesterol (HDL-c), low-density lipoprotein cholesterol (LDL-c), very low-density lipoprotein (VLDL-c) and triglycerides (TG) were determined by colorimetric quantification (AU5800 autoanalyzer, Coulter Beckman, USA). Plasma glucose was determined by photometry (AU5800 autoanalyzer, Coulter Beckman, USA).

### PBMCs isolation and culture

20 mL of peripheral blood anticoagulated with K_2_EDTA were immediately processed to obtain Peripheral Blood Mononuclear Cells (PBMCs) by a gradient-density centrifugation using Lymphoprep (Axis-Shield, Dundee, Scotland). Briefly, the anticoagulated blood was diluted with 15 mL PBS + K_2_EDTA 100 mM, the diluted blood was added slowly to a tube containing 15 mL of Lymphoprep, the tube was then centrifuged at 600 *g* for 30 min at 20 °C. PBMCs were separated and washed with PBS twice. Finally, the PBMCs were counted using a Neubauer chamber, aliquoted in a concentration of 1 × 10^7^ cells per vial and frozen in liquid nitrogen until use. For PBMCs culture, 1 × 10^7^cells were thawed, and resuspended in RPMI medium-1640 supplemented with 10% Fetal bovine serum (FBS), penicillin (100 U/mL), and streptomycin (100 µg/mL), (all from GIBCO™ Invitrogen, Corp., Carlsbad, CA, USA), at 37 °C in a humidified atmosphere of 5% CO_2_.

### Expression of receptors and ligands of the Fas and TRAIL pathways, activation markers and viral entry coreceptors in CD3^+^ CD4^+^ T-cells CD14^+^ CD4^+^ monocytes by flow cytometry

Immunophenotyping was performed using peripheral blood anticoagulated with EDTA. Anti-human monoclonal antibodies were used in the volume indicated by the manufacturer. An appropriate combination of fluorochromes was considered. In order to identify CD14^+^ CD4^+^ monocytes and their activation markers were used: PE-CD4 (clone: RPA-T4) and Alexa Fluor 700-CD14 (clone: 63D3); FITC-HLA-DR (clone: LN3); from BioLegend, San Diego, CA, and Alexa Fluor 700-CD80 (clone: L307.4), Alexa Fluor 700-CD86 (clone: FUN-1); both from BD, San Jose, CA. As well, APC-CD3 (clone: HIT3a) and PerCP-CD4 (clone: L200); Alexa Fluor 700-CD25 (clone: M-A251), Alexa Fluor 700-CD69 (clone: FN50), and FITC-CD38 (clone: HB-7); all from BioLegend, San Diego, CA; were used to identify CD3^+^ CD4^+^ T-cells. To identify the viral entry coreceptors were used Alexa Fluor 700-CXCR4 (clone: 12G5) and Alexa Fluor 700-CCR5 (clone: HEK/1/85a); from R&D, Minneapolis, MN and BioLegend, San Diego, CA; respectively. Finally, the antibodies FITC-CD95 (clone: DX2) and PE-CD78 (clone: NOK-1); both from BioLegend, San Diego, CA; Alexa Fluor 488-TRAIL (clone: 75402), Alexa Fluor 700 DR5 (clone: 71908); both from R&D, Minneapolis and FITC-DR4 (clone: DR-4-02) from Abcam, Cambridge, UK; were used to evaluate the expression of receptors and ligand of the Fas and TRAIL pathways.

Briefly, in each flow cytometry tube (BD, San Jose, CA), 100 µL of whole blood were placed, and the corresponding antibodies were added to each tube. Cells were incubated for 20 min at room temperature in the dark; afterward, erythrocytes were lysed with 900 1X lysis solution (FACS Lysing Solution; BD Biosciences, San Jose, CA, USA), washed and resuspended in PBS (Thermo Fisher Waltham, MA USA). Finally, the cells were acquired with the Attune^®^ NxT Acoustic Focusing Cytometer (Thermo Fisher Waltham, MA USA), at least 10,000 events were recorded for both cell populations. We assessed the expression by measuring the percentage of positive cells to each marker as well as the MFI (Mean Fluorescence Intensity). Flow cytometry data was analyzed using Attune Nxt Flow Cytometer Software version 2.6 (Thermo Fisher Waltham, MA USA).

### Apoptosis induction and blocking using Recombinant ligands and caspase inhibitor

PBMCs were thawed in RPMI with 10% FBS (both from GIBCO™ Invitrogen, Corp., Carlsbad, CA, USA), and seeded in 24 wells plate at a concentration of 2.5 × 10^5^ in 200 µL of complete medium. Caspase 8 was irreversibly inhibited with the addition of 100 µM of Caspase 8 inhibitor Z-IETD-FMK (R&D, Cat: 2163, MN, USA) and the cells were incubated for 2 h at 37 °C in a humidified atmosphere of 5% CO_2_ Afterwards, the recombinants ligands were added. Thus, the cells were incubated with 100 pg/mL Fas Ligand Membrane bound (Millipore CAT: 01-210, MA, USA) or 3 ng/mL TRAIL ligand (Millipore CAT: GF092, MA, USA) for 48 h at 37 °C in a humidified atmosphere of 5% CO_2_. After the incubations, the cells were collected and processed for apoptosis analysis by flow cytometry.

### Apoptosis analysis by flow cytometry

After apoptosis induction, PBMCs were collected, washed with PBS (Thermo Fisher Waltham, MA USA), and centrifuged at 300 g for 5 min. Supernatant was discarded and cell pellet was resuspended in 100 µL of PBS. For the identification of CD3^+^ CD4^+^ T-cell and CD14^+^ CD4^+^ monocytes, the cells were stained using the following monoclonal anti-human antibodies: APC-CD3 (clone: HIT3a), Alexa Fluor 700 CD14 clone: 63D3); both from BioLegend, San Diego, CA, and PerCP-CD4 (clone: L200) from BD, San Jose, CA. Cells with antibodies were incubated at room temperature for 20 min. Annexin V/PI staining was used to distinguish events in early and late apoptosis. Thus, cells were washed with PBS and resuspended in 100 µL of Annexin V Buffer and stained using 5µL of Annexin V-Alexa 488 and 1µL of Propidium Iodide (PI), using the Alexa Fluor 488 Annexin V/ Dead Cell Apoptosis Kit (Thermo Fisher, Cat: V13242, Waltham, MA, USA) and were incubated at room temperature for 15 min. Finally, 400 µL of Annexin V Buffer were added to the tubes, and the cells were acquired by the Attune^®^ NxT Acoustic Focusing Cytometer ((Thermo Fisher Waltham, MA USA). At least 10,000 events were acquired for both cell populations, data was analyzed using Attune Nxt Flow Cytometer Software version 2.6 (Thermo Fisher Waltham, MA USA).

### Inflammatory cytokines

A Multiplex bead-based immunoassay (LEGENDplex Human Inflammation Panel; BioLegend, San Diego, CA, USA) was used to quantify cytokine levels (TNF-α, IL-1β, IL-8 and IL-18) in serum of PLWHIV and HIV- controls following the manufacturer´s protocol. 300 events per analyte were acquired using the Attune Nxt Flow Cytometer Software version 2.6 (Thermo Fisher Waltham, MA USA). The files were analyzed utilizing LEGENDplex Data Analysis Software v8 (BioLegend, San Diego, CA, USA). Results represent the concentration expressed in pg/mL.

### Statistical analysis

Statistical analysis and graphical representations were performed in SPSS v20 (IBM Corp., NY, USA) and GraphPad Prism 6 (La Jolla, CA, USA); respectively. Depending on their distribution, data are presented as mean ± standard deviation and median (interquartile range). Parametric and non-parametric tests for independent variables were used accordingly on the data distribution. Mann Whitney U, Student’s T and Spearman test, were used. For the apoptosis induction between cell populations, we performed the Friedman test for multiple treatments in a non-parametric population. A value of *p* < 0.05 was considered significant.

## Results

### Demographic and clinical data

Demographic and clinical data of participants are shown in Table 1. The viral load mean in the PLWHIV was 25,563 copies/mL. The median of absolute count of CD4^+^ and CD8^+^ T-cells was 470 (386–636) and 1135 (795–1438) cells/µL; respectively. The median CD4^+^/CD8^+^ ratio was 0.39 (0.32–0.67). Finally, they reported a sexual life onset of 17 ± 2.4 years on average and an infection time of 7.9 ± 5.3 months (Table 1). Regarding to biochemical profile no statistical differences were found (Table 2).

**Table 1:** Demographic data of PLWHIV and HIV- controls

Variable	HIV- *n* = 14	PLWHIV *n* = 14
Age (years); mean ± SD	27.88 ± 6.46	28.73 ± 6.72
HIV-1 RNA Viral load; mean ± SD	-	25,563.84 ± 18336.51
CD4^+^T-cells count (cells/µL); median (IQR)	-	470 (386.25-636.25)
CD8^+^ T-cells count (cells/µL); median (IQR)	-	1134.5 (795-1438.25)
CD4^+^/CD8^+^ ratio; median (IQR)	-	0.39 (0.32–0.67)
Start of sexual activity (years); mean ± SD	-	17.14 ± 2.41
Time of infection (months); mean ± SD	-	7.88 ± 5.3

**Table 2:** Clinical data of PLWHIV and HIV- controls. Student’s t-test. All values are expressed as mean and standard deviation

Variable (mg/dL) (mean ± SD)	HIV- *n* = 14	PLWHIV *n* = 14	*p* value
Cholesterol	190 ± 28.73	167.62 ± 32.05	0.86
Triglycerides	102.38 ± 53.34	126 ± 48.58	0.485
HDL	47.46 ± 8.87	36 ± 6.12	0.268
LDL	122.15 ± 23.34	102.85 ± 37.42	0.402
VLDL	20.54 ± 10.6	28.77 ± 15.01	0.077
Glucose	82.08 ± 7.42	93.85 ± 5.55	0.297

### Expression of ligands and receptors of Fas and TRAIL pathways

**Fig. 1:**
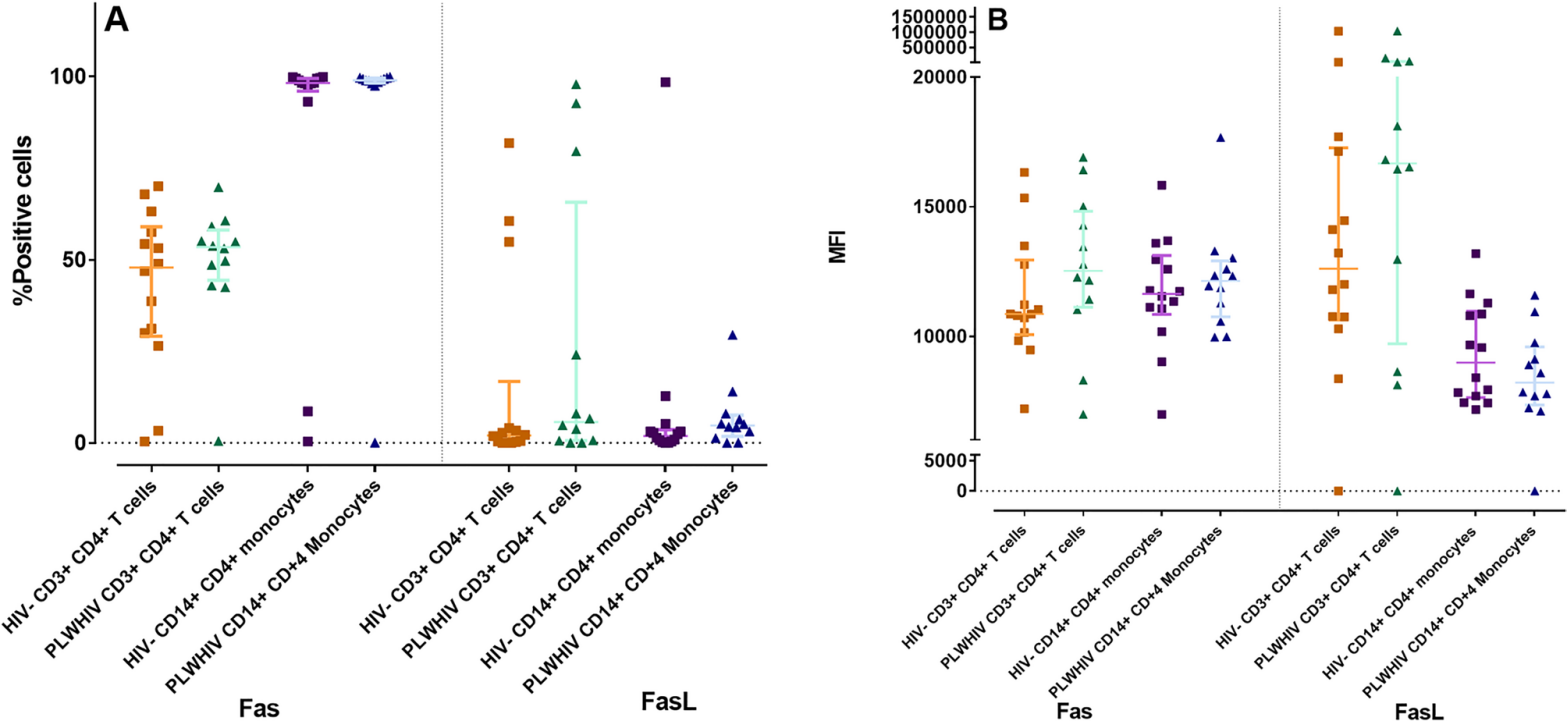
Fas Pathway. Differences in the percentage of positive cells (**A**) and MFI (**B**) of the Fas Pathway markers in CD3^+^ CD4^+^ T-cells and CD14^+^ CD4^+^ monocytes from PLWHIV and HIV- controls. Based on the data distribution, we used the Mann Whitney U test. *p *< 0.05*; ** *p* < 0.01

**Fig. 2:**
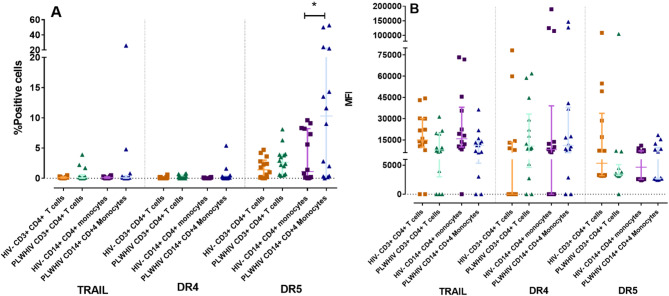
TRAIL Pathway. Differences in the percentage of positive cells (**A**) and MFI (**B**) of the TRAIL Pathway markers in CD3^+^ CD4^+^ T-cells and CD14^+^ CD4^+^ monocytes from HIV + patients and HIV- controls. Based on the data distribution, we used the Mann Whitney U test. *p *< 0.05*; ** *p* < 0.01

The first objective was to determinate by flow cytometry, the expression of Fas/FasL and DR4/DR5/TRAIL in CD3^+^ CD4^+^ T-cells and CD14^+^ CD4^+^ monocytes from PLWHIV and HIV- controls. Cell expression percentage as well as the MFI are shown in Table 3; Fig. 1, and Fig. 2.

Fas and FasL showed no significant differences, however there was a slight increase in the expression percentage and MFI of Fas and FasL in CD3^+^ CD4^+^ T-cells from PLWHIV; the same behavior was observed in the case of FasL percentage in CD14^+^ CD4^+^ monocytes from PLWHIV, (Table 3, and Fig. 1A).

**Table 3:** Expression percentage and MFI of Fas and TRAIL pathways receptors and ligands in CD3^+^ CD4^+^ T-cells and CD14^+^ CD4^+^ monocytes from PLWHIV and HIV- controls. All values are expressed as median and interquartile range. Based on the data distribution, we used the Mann Whitney U test

	CD3^+^ CD4^+^ T-cells			CD14^+^ CD4^+^ Monocytes		
Cell death pathways Positive cells	HIV- (*n* = *14*) mean (IQR)	PLWHIV (*n* = *14*) mean (IQR)	*p* value	HIV- (*n* = *14*) mean (IQR)	PLWHIV (*n* = *14*) mean (IQR)	*p* value
TRAIL %	0.1(0–0.2)	0.1 (0–0.2)	0.567	0 (0–0)	0 (0–0.1)	0.601
TRAIL MFI	14,915 (9939.50–30077.75)	7932 (3291–14593)	0.053	15,838.5 (9998.5–38076.75)	11,758 (6689–15451.5)	0.089
DR4%	0 (0–0.2)	0 (0–0.25)	0.361	0.05 (0–0.1)	0.1 (0–0.1)	0.207
DR4 MFI	0 (0–13069)	15,015 (8746–37055)	0.082	8276 (0–13732)	14,348 (7921–38947)	0.356
DR5%	1.4 (0.2–2.8)	2.65 (0.65–3.85)	0.108	1.10 (0.10–8.20)	5.90 (0.35–13.90)	*< 0.05*
DR5 MFI	5320 (3188–28593)	3387 (3223–5221)	0.462	4658 (2586–7159)	2709 (2619–7444)	0.383
Fas %	47.9 (30–57.6)	53 (45.8–53.45)	0.411	98.3 (97–99.5)	99 (98.3–99.5)	0.486
Fas MFI	10,857 (10144–12764)	12,525 (11221–14641)	0.136	11,637 (11068–12958)	10,927 (12134–12802)	0.643
FasL %	2.05(0.3–4.1)	5.7 (0.65–51.85)	0.180	1.9 (0.4–3.1)	4.75 (2.2–7.2)	0.142
FasL MFI	12,608 (10752–17133)	16,671 (10797–37939)	0.269	8981 (7692–10856)	8211 (7465–9430)	0.368

**Fig. 3:**
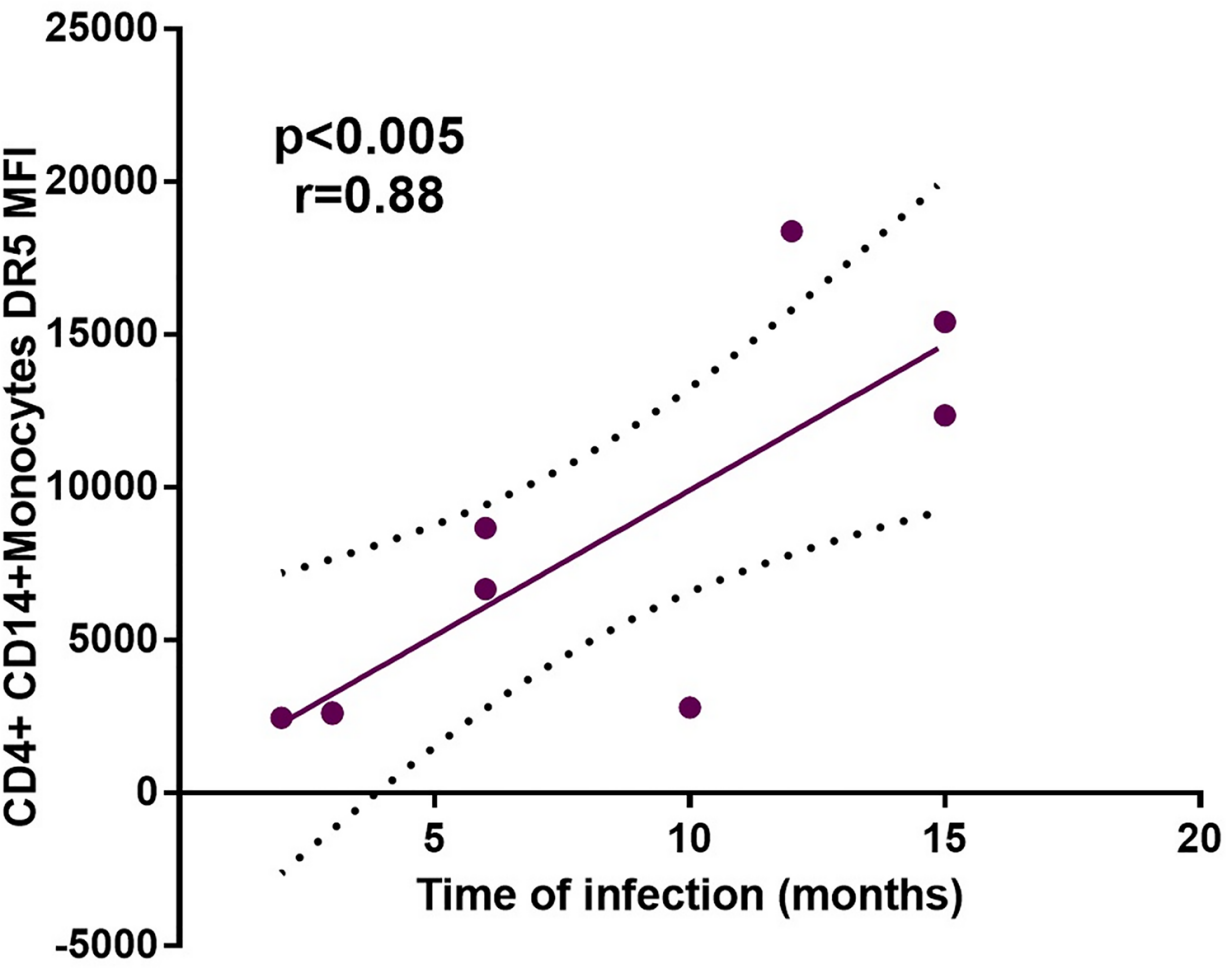
Correlation between time of HIV infection of HIV + patients and the MFI of DR5 receptor from TRAIL pathway in CD14^+^ CD4^+^ monocytes. Spearman correlation was used

Regarding the TRAIL receptors (DR4 and DR5) and TRAIL ligand (TRAIL), the expression percentage of TRAIL in both CD3^+^ CD4^+^ T-cells and CD14^+^ CD4^+^ monocytes was extremely low; however, in both cell populations from PLWHIV the amount of TRAIL expressed per cell was lower than expressed in HIV- controls, as indicated by the MFI, (Table 3; Fig. 2B). Additionally, an increase was observed in the MFI of DR4 in PLWHIV in both cell populations, however this increase was not statistically significant. Interestingly, it was found a significantly higher expression (*p* < *0.05*) of DR5 in CD14^+^ CD4^+^ monocytes from PLWHIV, while for CD3^+^ CD4^+^ T-cells there was only a slight increase of this receptor, without this difference being significant in comparison to HIV- controls (Table 3; Fig. 2A). Besides, with the data obtained so far, a positive correlation (*r* = 0.88, *p* < 0.005) was found between the time of HIV infection and the MFI of DR5 in CD14^+^ CD4^+^ monocytes from PLWHIV, (Fig. 3).

### Coreceptors CXCR4 and CCR5

**Fig. 4:**
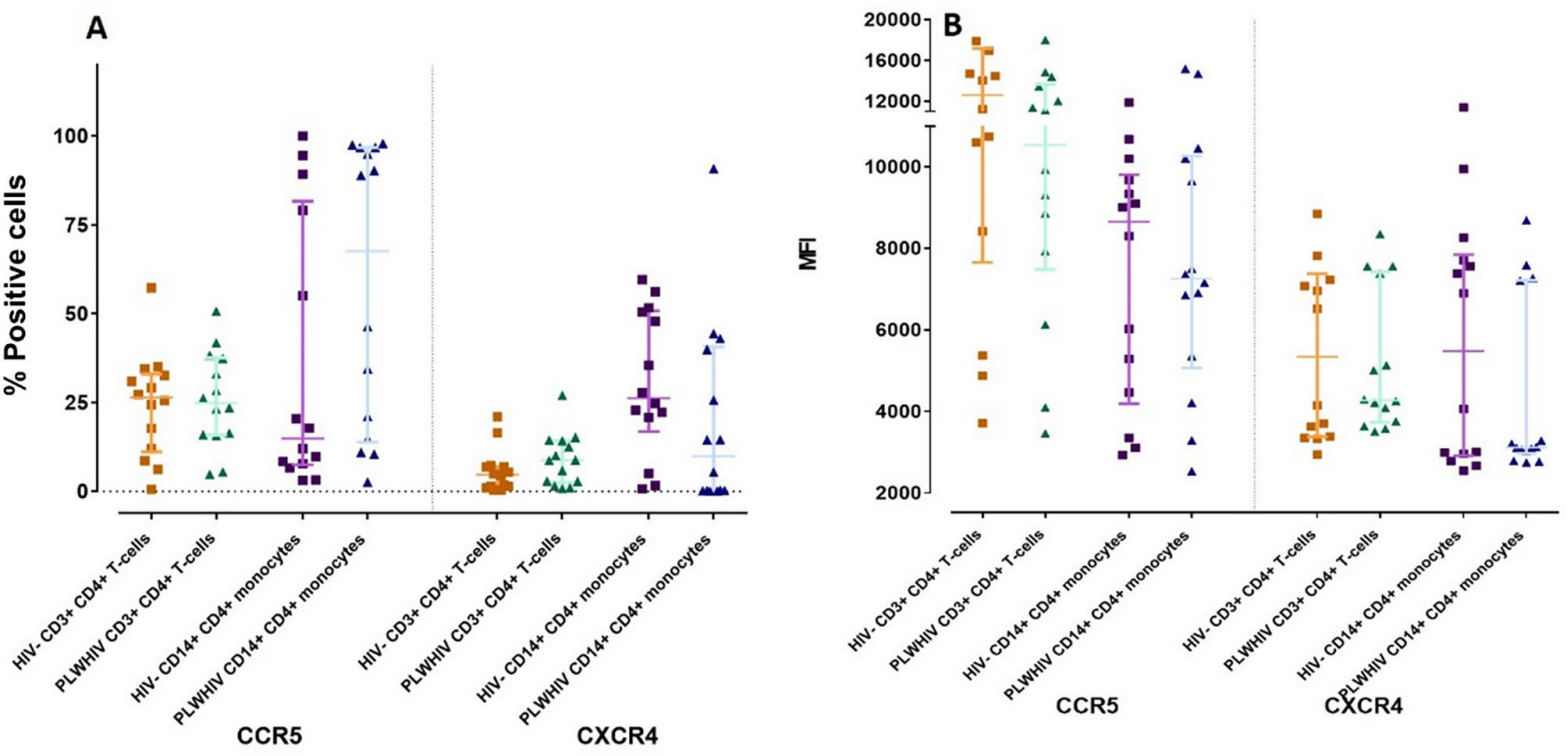
Positive cells percentage (**A**) and MFI (**B**) of CCR5 and CXCR4 HIV entry coreceptors in CD3^+^ CD4^+^ T-cells and CD14^+^ CD4^+^ monocytes from PLWHIV and HIV- controls. Based on the data distribution, we used the Mann Whitney U test. *p *< 0.05*; ** *p* < 0.01

It has been documented that signaling through CXCR4 and CCR5 coreceptors are a potent apoptotic stimulus since they can up regulate the expression of ligands and receptors of Fas and TRAIL pathways [2]. The next objective was the determination CCR5 and CXCR4 expression in CD3^+^ CD4^+^ T-cells and CD14^+^ CD4^+^ monocytes from PLWHIV and HIV- controls. The results of the percentage of expression and MFI are shown in Table 4; Fig. 4; respectively.

**Table 4:** Expression percentage and MFI of CCR5 and CXCR4 HIV entry coreceptors in CD3^+^ CD4^+^ T-cells and CD14^+^ CD4^+^ monocytes from PLWHIV and HIV- controls. All values are expressed as median and interquartile range. Based on the data distribution, we used the Mann Whitney U test

	CD3^+^ CD4^+^ T-cells			CD14^+^ CD4^+^ Monocytes		
Correceptorpositive cells	HIV- (*n* = *14*)	PLWHIV (*n* = *14*)	*p* value	HIV- (*n* = *14*)	PLWHIV (*n* = *14*)	*p* value
CCR5%	26.45 (12–32.6)	27.3 (19.55–37.85)	0.854	14.90 (7.8–79.1)	89.60 (27.8–96.8)	0.098
CCR5 MFI	12,646 (8421–16,954)	10,650 (8397-13,929)	0.312	8658 (4468–9679)	7432 (6134–10332)	0.854
CXCR4%	4.70 (1.4–6.9)	7.30 (2.15–13.35)	0.161	9.95 (0.3–39.9)	26.25 (12.9–51.1)	0.060
CXCR4 MFI	5338 (3387–7236)	4234 (3702–7473)	0.748	5484 (2962–7710)	3120 (2927–7240)	0.613

Although no significant differences were found, a slight increase in the expression percentage of CCR5 and CXCR4 in CD3^+^ CD4^+^ T-cells, was observed. This increase was more evident in CD14^+^ CD4^+^ monocytes (*p* = 0.098 and *p* = 0.060; respectively) from PLWHIV compared to HIV- controls, (Table 4; Fig. 4A).

**Fig. 5:**
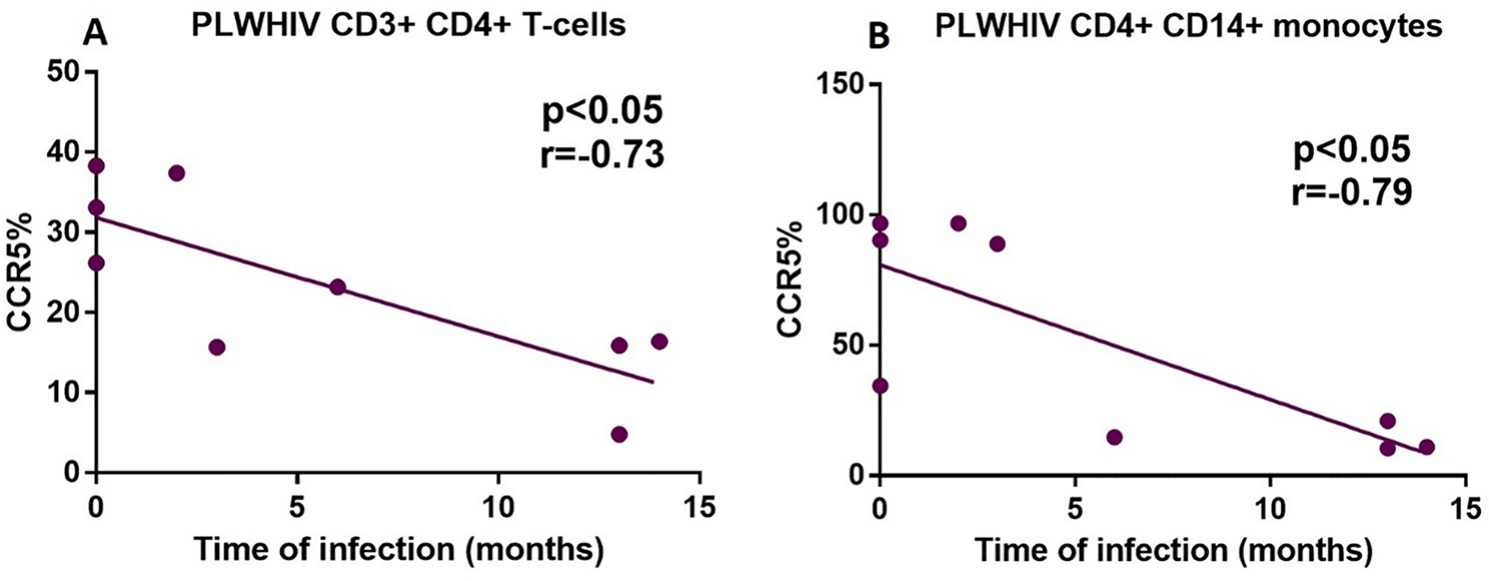
Negative correlation between time of HIV infection and CCR5 coreceptor percentage of positive cells in both CD3^+^ CD4^+^ T-cells and CD14^+^ CD4^+^ monocytes from PLWHIV. Based on the distribution, we used the Spearman correlation, with a significant p value of *p* < 0.05

**Fig. 6:**
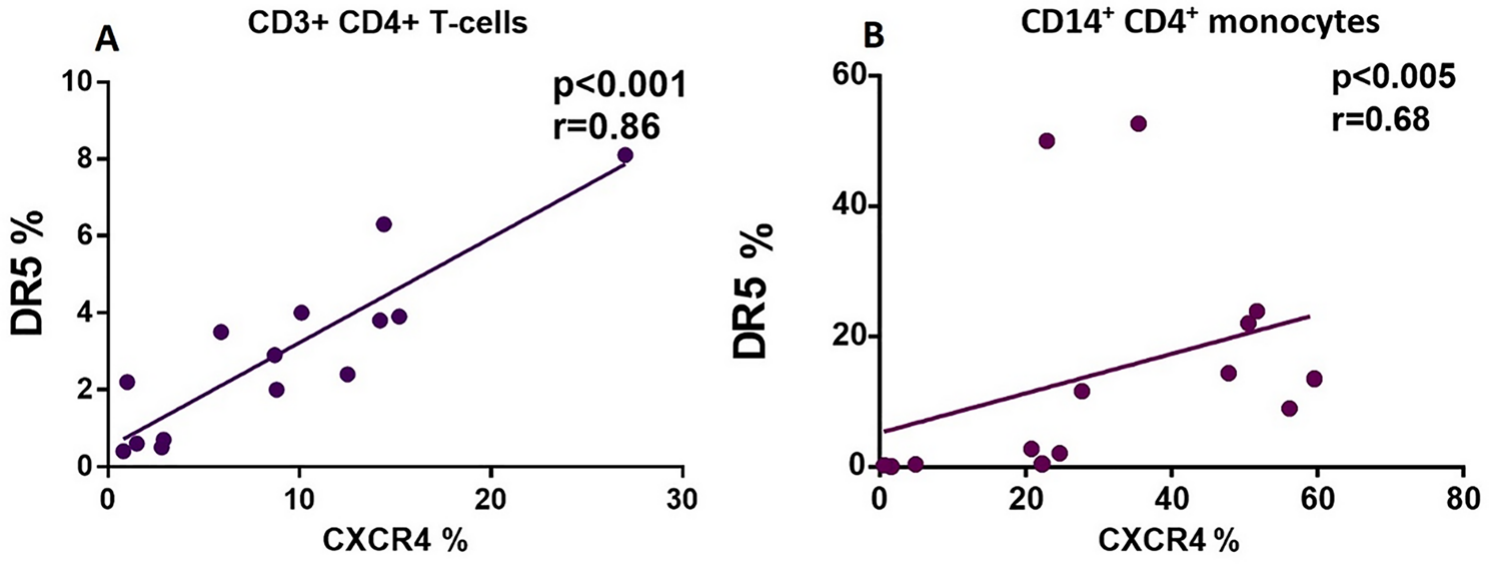
Positive correlation between DR5 receptor positive cells and CXCR4 coreceptor positive cells in both CD3^+^ CD4^+^ T-cells and CD14^+^ CD4^+^ monocytes from PLWHIV. Based on the distribution, we used the Spearman correlation, with a significant p value of *p* < 0.05

Additionally, in Fig. 5A and B, it can be seen a negative correlation between time of HIV infection and the expression percentage of CCR5 in CD3^+^ CD4^+^ T-cells as well as CD14^+^ CD4^+^ monocytes from PLWHIV (*r* = − 0.73; *p* < 0.05 and *r* = − 0.79; p *< 0.05*); respectively. This indicates a decrease in CCR5 in relation to the progression of the disease. With respect to the percentage of expression of CXCR4, a positive correlation with the DR5 in CD3^+^ CD4^+^ T-cells (*r* = 0.86; *p* < 0.001) and CD14^+^ CD4^+^ monocytes (*r* = 0.68; *p* < 0.005) from PLWHIV was observed, (Fig. 6).

### Activation cell markers in CD3^+^ CD4^+^ T-cells and CD14^+^ CD4^+^ monocytes

**Fig. 7:**
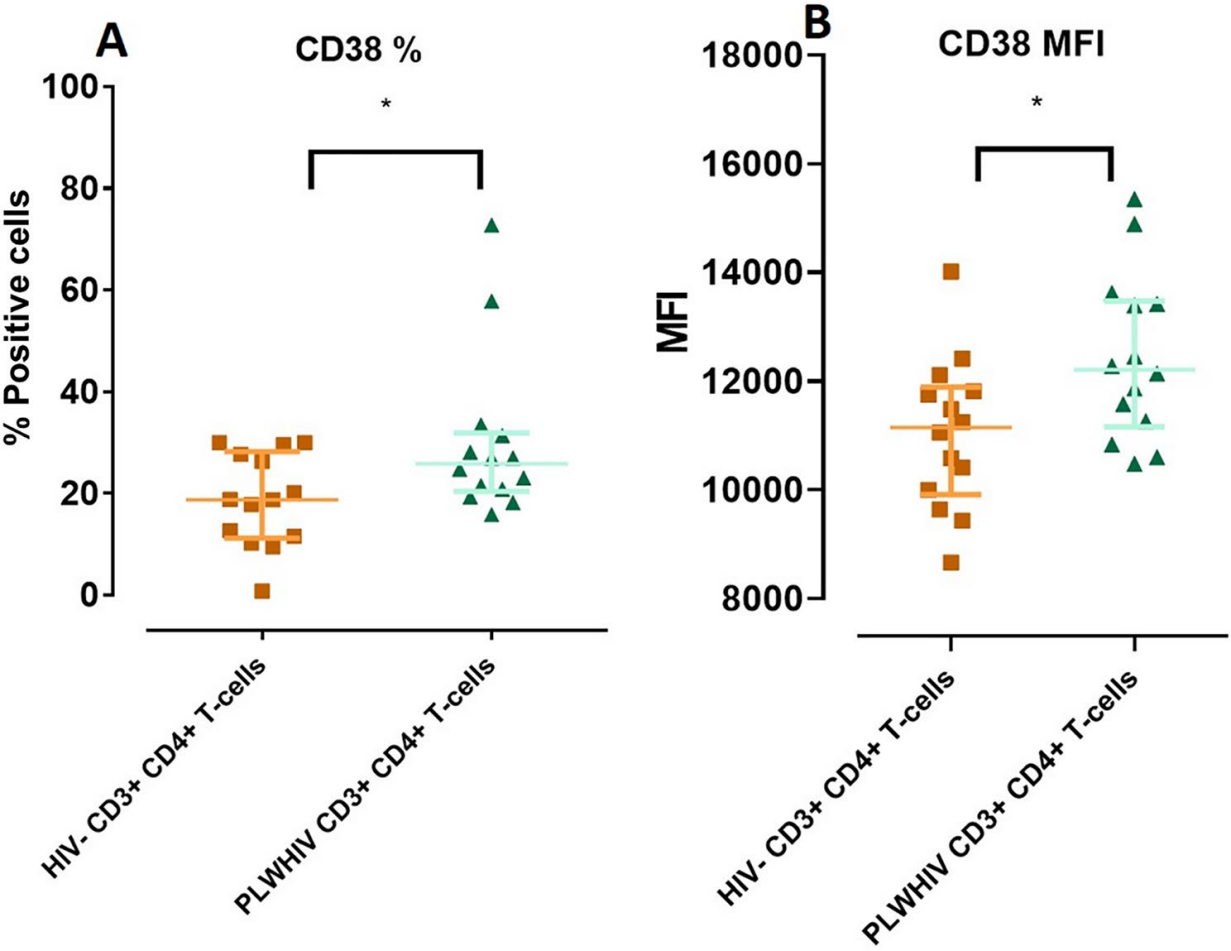
Differences in the percentage of positive cells and MFI of CD38 between PLWHIV and HIV- CD3^+^ CD4^+^ T-cells. Based on the data distribution, we used the Mann Whitney U test. *p *< 0.05*

A state of immune hyperactivation is characteristic of HIV infection and is associate with cell death by apoptosis. The activation markers CD25, CD69 and CD38 for were evaluated in CD3^+^ CD4^+^ T-cells and CD80, CD86 as well as HLA-DR were measure measured in CD14^+^ CD4^+^ monocytes. We found no significant differences in the activation markers measured in monocytes (data not shown). Regarding to CD3^+^ CD4^+^ T-cells from PLWHIV, only CD38 showed a significant increase (*p* < *0.05*) in percentage of expression as wells as in MFI, respect to HIV- controls, (Fig. 7).

### Cytokine determination

**Fig. 8:**
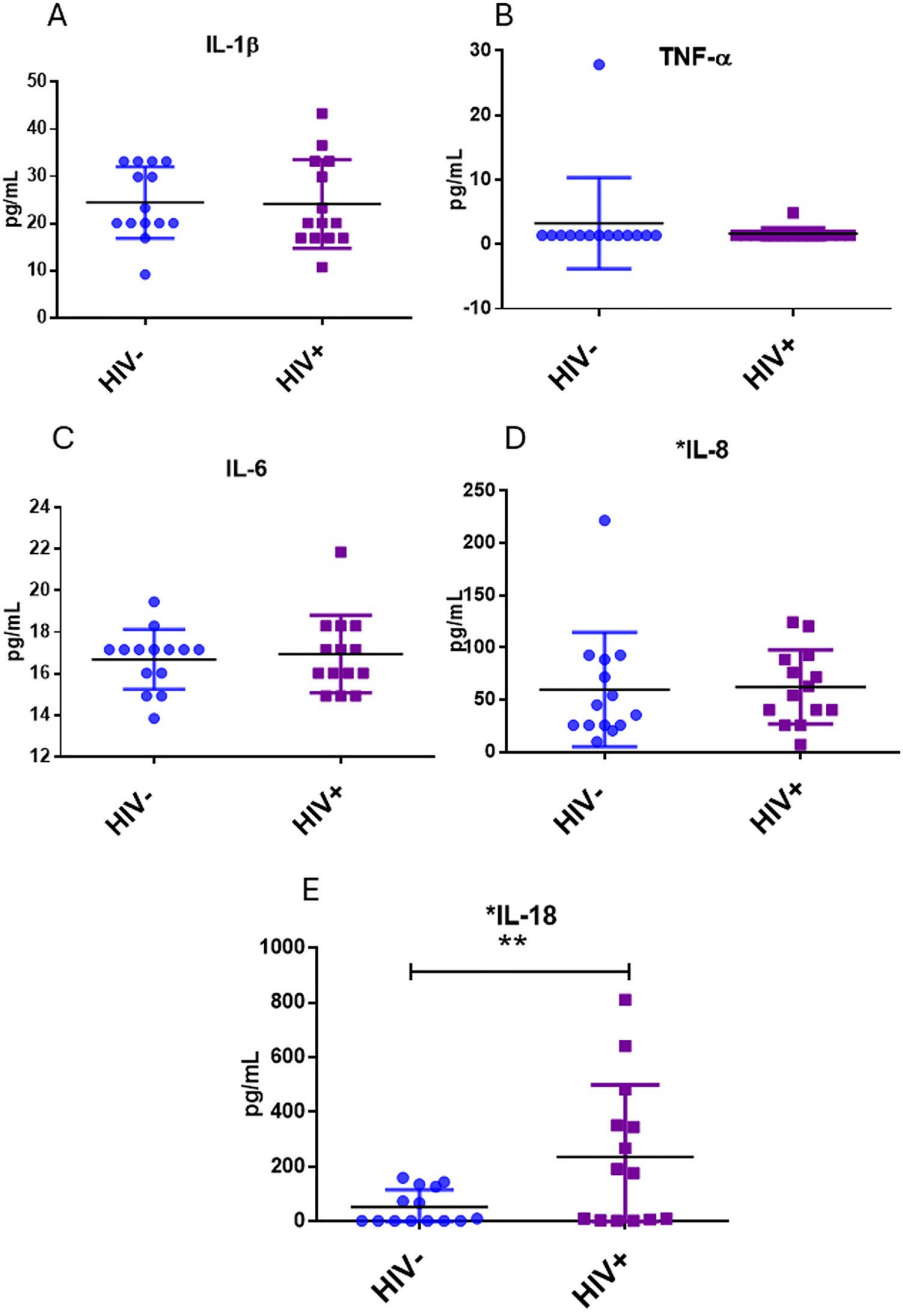
Inflammatory cytokines measured in sera of PLWHIV vs. HIV- controls. Based on the data distribution, we used the Mann Whitney U test. *p < 0.05

**Fig. 9:**
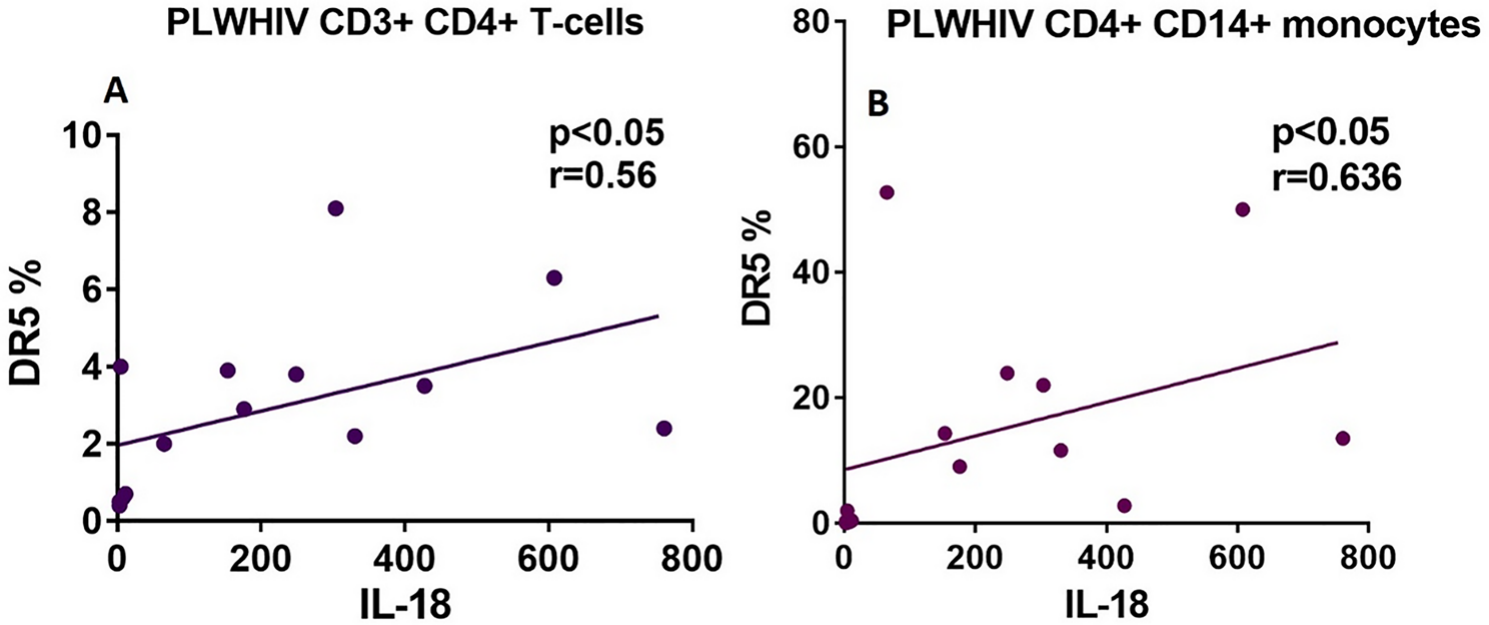
Positive correlation between DR5 receptor positive cells percentage and IL-18 quantity in both CD3^+^ CD4^+^ T-cells and CD14^+^ CD4^+^ monocytes from PLWHIV. Based on the distribution, we used the Spearman correlation, with a significant p value of *p* < 0.05

Elevated concentrations of sera inflammatory cytokines are indicators of systemic inflammation with potential to promote apoptosis in HIV infection. In this sense, IL-1β, TNFα, IL-6, IL-8 and IL-18 were evaluated. Only IL-18 was significantly increased in PLWHIV compared to HIV- controls (*p* < *0.05*), (Fig. 8E), while the other cytokines were not (Fig. 8A-D). Furthermore, IL-18 showed a significant positive correlation with the percentage expression of DR5 in CD3^+^ CD4^+^ T-cells (*r* = 0.56; *p* < 0.05) and CD14^+^ CD4^+^ monocytes (*r* = 0.636; *p* < 0.05), (Fig. 9).

### Apoptosis induction and Inhibition of FAS and TRAIL pathways

**Fig. 10:**
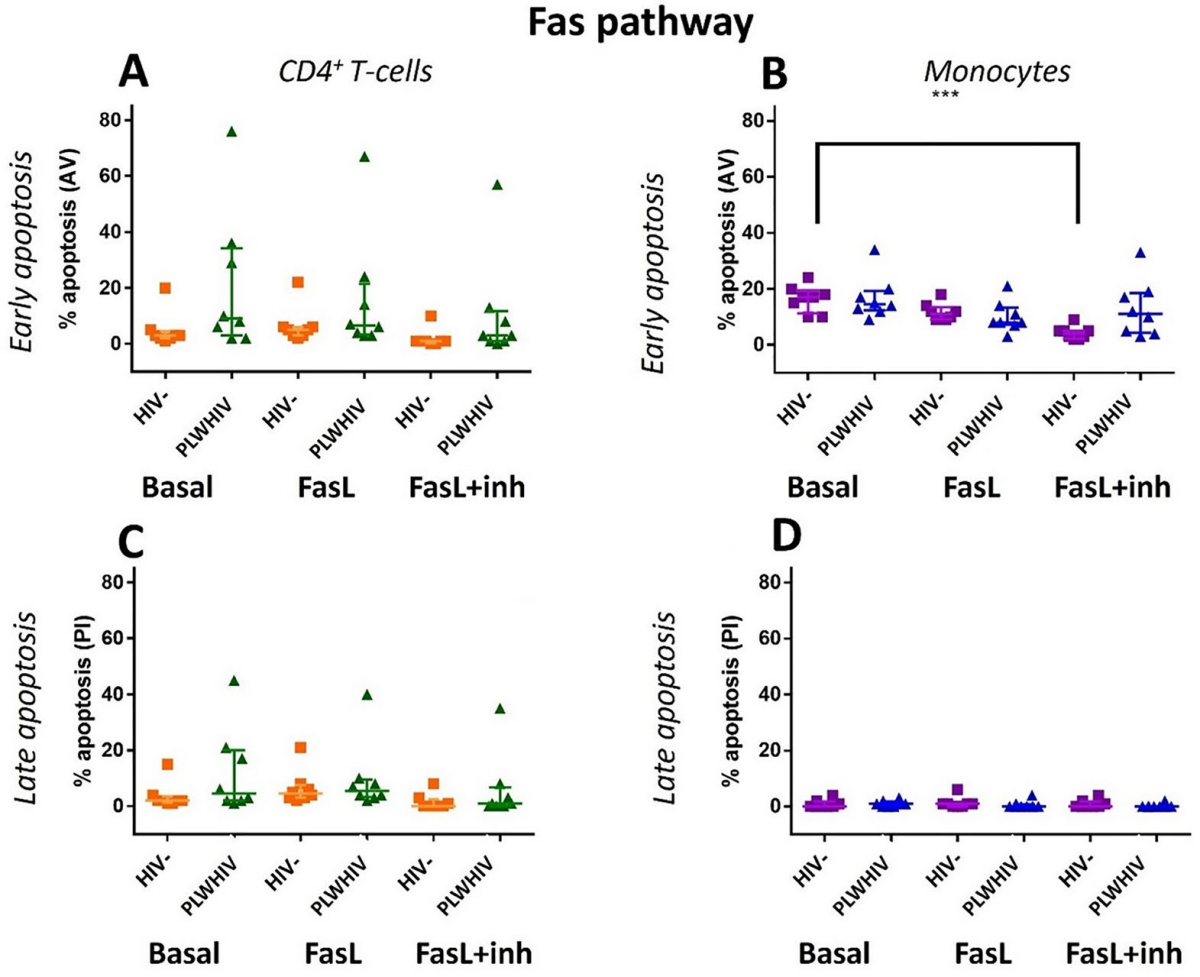
Early and late apoptosis in Fas pathway. A and C graphs: CD3^+^ CD4^+^ T-cells, B and D graphs: CD14^+^ CD4^+^ monocytes. The basal cells were incubated without treatment, the FasL cells were incubated with the recombinant ligand and the FasL + inh were incubated with the Caspase 8 inhibitor and the recombinant ligand. For the group comparisons we used the Friedman Test. *p < 0.05; ** *p* < 0.01 and *** *p* < 0.001, non-significant differences were found between all comparisons except basal and FasL + inh HIV- monocytes in early apoptosis

**Fig. 11:**
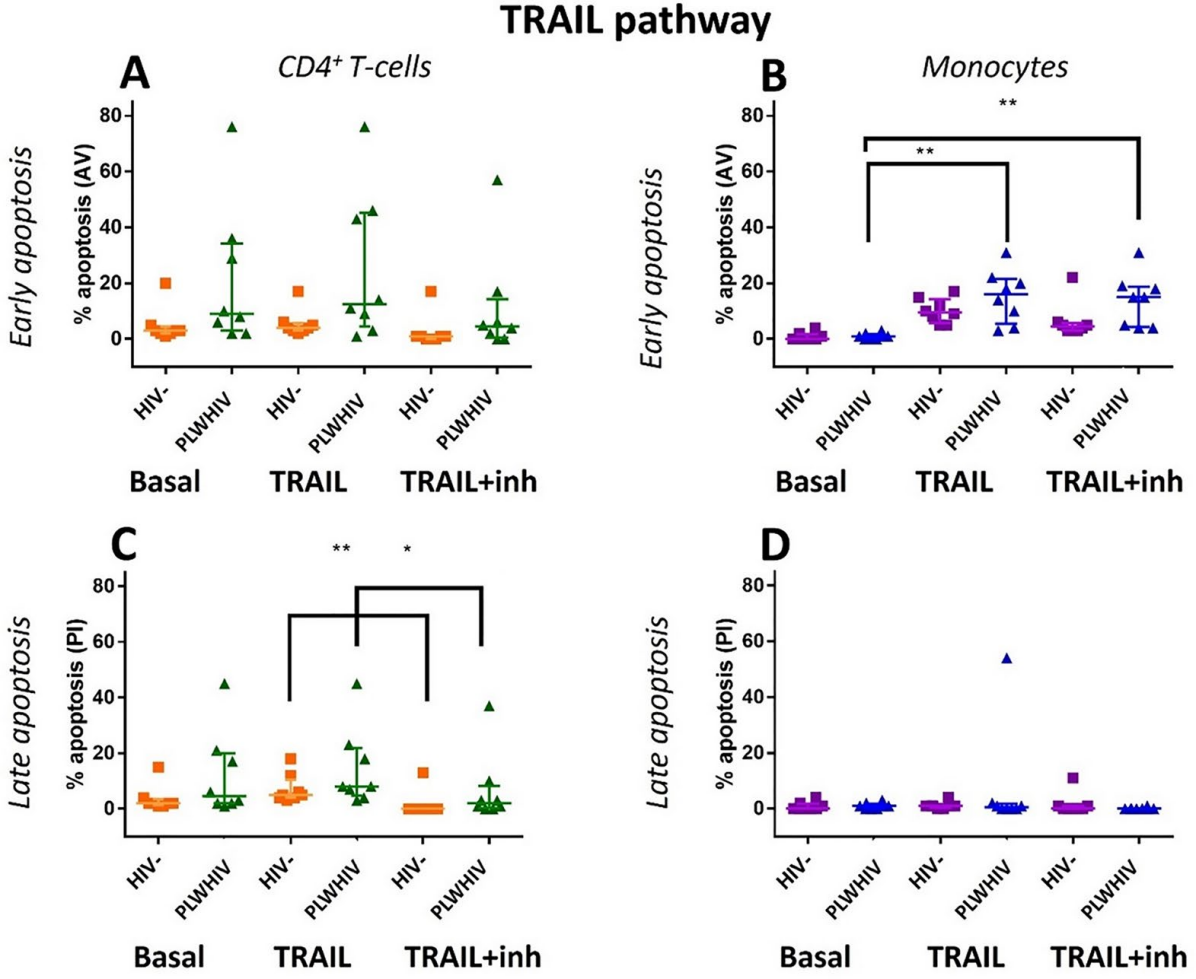
Early and late apoptosis in TRAIL pathway. **A** and **C** graphs: CD3^+^ CD4^+^ T-cells, **B** and **D** graphs: CD14^+^ CD4^+^ monocytes. The basal cells were incubated without treatment, the TRAIL cells were incubated with the recombinant ligand and the TRAIL + inh were incubated with the Caspase 8 inhibitor and the recombinant ligand. For the group comparisons we used the Friedman Test. *p *< 0.05*; ** *p* < 0.01 and *** *p* < *0.001.* Non-significant differences were found between all comparisons except: early apoptosis in between basal and TRAIL treated and basal and TRAIL + inh treated monocytes of PLWHIV, and late apoptosis of TRAIL and TRAIL + inh CD4^+^ T-cells in HIV- controls

Finally, to evaluate the sensibility to apoptosis triggered by Fas and TRAIL pathways it was carried out an in vitro stimulation of the PBMCs derived from PLWHIV and HIV- controls. Figures 10 and 11 show that the HIV- control group, had a homogeneous behavior and, the percentage of apoptosis never exceeded 30% for Fas and TRAIL pathways. In CD3^+^ CD4^+^ T-cells, no significant differences were found in the induction and inhibition of the Fas pathway. However, it was observed that PLWHIV cells have a slight increase in early and late apoptosis compared to HIV- controls (Fig. 10A and C). Only in CD14^+^ CD4^+^ monocytes from HIV- control was observed that there was a significant decrease in early apoptosis between the basal and inhibition treatment (*p* < *0.001)*, (Fig. 10B). In the case of the TRAIL pathway, particularly in the CD3^+^ CD4^+^ T-cells derived from PLWHIV and HIV- controls there was a significant decrease in late apoptosis, almost to the basal level, which was more evident in HIV- controls, (Fig. 11C). Interestingly, in CD14^+^ CD4^+^ monocytes from PLWHIV, early apoptosis was significantly increased when the recombinant ligand is added, and this effect was maintained even when the caspase 8 inhibitor was added, (Fig. 11B).

## Discussion

Fas death pathway is considered the most important in CD4^+^ T-cells depletion in PLWHIV. It has been extensively documented that the FasL is augmented in CD4^+^ T-cells, monocytes, macrophages and NK cells [9, 10]. In the present study it was found that there was a non-significant increase in the FasL expression on CD3^+^ CD4^+^ T-cells and CD14^+^ CD4^+^ monocytes from PLWHIV. In a study performed by Sloand in 1997, was observed a similar difference in FasL expression, however, they considered all the positive T-cells without making a distinction between CD4^+^ and CD8^+^ T-cells and since it has been documented that CD8^+^ T-cells have a notably augmented expression of FasL, this could explain the discrepancy of our study with the aforementioned study [20]. In concordance with existing literature, our results confirm that FasL expression was higher in lymphocytes compared to monocytes and higher in CD^4+^ lymphocytes of PLWHIV compared to negative subjects, however this was not statistically significant, possibly due to the sample size. Furthermore, elevated levels of sFas and sFasL as well as a positive correlation of sFasL with the viral load has been previously reported [21, 22], undoubtedly a marker of interest in PLHIV before ART. Thus, in PLWHIV an increased FasL turnover towards soluble sFasL should be considered [21].

The expression and MFI of Fas was elevated in both study groups with a non-significant difference. However, it was observed that there was a subtle elevation in PLWHIV compared to HIV- controls. Sloand et al. observed something similar, finding an expression of up to 60% in T-cells from PLWHIV. Additionally, in Sloand study included subjects with a CD4^+^ T-cells count < 200 cells/µL, which showed a broader receptor expression compared to subjects who had a higher cell count, data in accordance with our results since PLWHIV in this study had more than 200 cells/µL [20]. Adding to this, the population from Sloand’s study was larger, so we can hypothesize that an increase in sample size could transform the trend and improve the p-value and thereby achieve statistical significance between both groups. Thus, cells from PLWHIV would be more susceptible to apoptosis through the Fas pathway due to the expression of both molecules, Fas and specially FasL on these cells, particularly in monocytes. It is important to consider that the expression of receptors and ligands of death pathways, is not necessarily correlated to apoptosis sensitivity, the function can be modified by inhibitory mechanisms, such as: soluble protein blocking of ligands or receptors, among them Bcl-2 family inhibitors. Additionally, it has been previously described that some HIV viral proteins have both pro and anti-apoptotic effects, which can affect the extrinsic pathway sensibility independently of receptor expression. HIV protease as well as Tat and Env, can cause Caspase 8 excision [23], as well as the increase of pro apoptotic proteins such as Bax [24] and the decrease of FLIP expression [25, 26]. Regarding functional studies of Fas and TRAIL pathways, we did not found an increased in apoptosis sensibility in PLWHIV, contrary to previous reports [20, 27]. However, the methodologies used were different. Estaquier, et al. performed light microscopy with chromatin condensation, and they used purified CD4^+^ and CD8^+^ T-cells instead PBMCs [27]. Sloand et al. used DNA quantification by cytometry to evaluate the functionality of apoptotic pathways, and additionally, the study population had a low CD4^+^ T-cell count, which would make them more sensitive to the inducted apoptosis [20].

The TRAIL pathway has been less studied in the context of HIV infection. It has been observed that the use of recombinant ligand can cause apoptosis only to infected cells without significantly affecting the functionality of uninfected T-cells [28]. It should be noted that this is the first study that evaluates the expression of TRAIL in cells from PLWHIV before ART, and interestingly it was observed a significant elevation of DR5 expression on CD14^+^ CD4^+^ monocytes and CD3^+^ CD4^+^ T-cells of PLWHIV although in the last population there was only an increasing trend. In the case of DR4 and ligand TRAIL, the expression turned out to be almost null in both study groups. Previously, it has been described a high expression of TRAIL/-Apo2L as well as the decoy receptor 2 (DCR2) and DR5 at level of RNA and surface expression in CD4^+^ and CD8^+^ T-cells from PLWHIV [15]. Additionally, in vitro and ex vivo models have shown that the viral infection or viral proteins as gp120 and Tat induce the expression of TRAIL, as well as the four receptors of TRAIL on T-cells, monocytes, DC and PBMCs [10, 15, 17, 29, 30]. More recently, it has been identified a TRAIL splice variant that is produced by HIV infected and non-infected cells with the capacity of to generate resistance to apoptosis via TRAIL since it binds and directs proteasomal degradation to DR1 and DR2 receptors [31]. It has been previously described that in T-cell from elite controllers [19] as well as slow progressors [32] the expression of DR5 is diminished and even similar to the control group (this could be to the low rate of infection found in this subjects). In the case of our population of PLWHIV it is not possible to consider them slow progressors, keeping in mind the estimated time of infection in them is below two years and that to be included in this group they would have to keep a CD4^+^ T-cells count above 350 for more than 10 years without treatment and maintain a low viral load. Observations made by Herbeuval et al. consider DR5 as a possible marker of HIV infection [29], hypothesis that is supported by this study since DR5 expression was significantly increased in CD14^+^ CD4^+^ monocytes of PLWHIV and that its expression was positively correlated with the time of infection. Regarding the functional assays, we did not find an increase in the apoptosis induced by the TRAIL ligand. Contrary to the results reported by Lum in 2001 [15], who with a similar methodology and Hoechst stain, reported an elevated induced apoptosis in cells from PLWHIV, however, they do not refer to the CD4 count they considered. As mentioned previously, this parameter is fundamental in sensitivity to apoptosis.

Regarding the entry coreceptors, there was any significant difference, just an increased expression of CCR5 on PLWHIV monocytes. However it was found a negative correlation between this coreceptor expression in both CD3^+^ CD4^+^ T-cells and CD14^+^ CD4^+^ monocytes from PLWHIV and the time of infection, which agrees with the natural tropism history in which, the R5 tropism is gradually lost due to the depletion of the cells which express CCR5 receptor [33]. In studies performed with both lymphocytic cell lines and primary lymphocytes cultures, using as stimulus the viral protein gp120 directed to CD4 and CXCR4 was reported that, binding of the glycoprotein with the coreceptor CXCR4 increased the expression of the TRIAL receptor, while the single binding with CD4 was not capable of inducing this phenomenon [34]. In a similar work, with cells that selectively expressed CD4 and CXCR4 proteins with the glycoprotein gp120, it was observed that binding of the gp120 to CXCR4 caused caspase dependent apoptosis, assessed using an inhibitor caspases 1, 3, 7 and 8, which blocked apoptosis induced by gp120 union [35]. Thus, hypothesizing that the expression and stimulation of CXCR4 is necessary to modify the expression of TRAIL receptors and the activity of TRAIL pathway. In this sense, in the context of HIV infection, is known that the CXCR4 expression is late, and associated with X4 strains and a rapid depletion of CD4^+^ T-cells in approximately 50% of infected individuals. This event may be associate with the gain in the expression and function of the TRAIL pathway in late stages of HIV infection, hypothesis that could support the inactivity of the pathway reported in this study [36]. Thus, several mechanisms of proapoptotic effects have been proposed for the glycoprotein gp120, which include the upregulation of Fas, FasL, TRAIL and TNF-α expression [10]. Regarding our study, even if CXCR4 was not found significantly elevated, probably due to the inclusion of PLWHIV in early stage of infection, a positive correlation between CXCR4 and DR5 expression (*p* < 0.001) in both CD3^+^ CD4^+^ T-cells (*r* = 0.86; *p <* 0.001) and CD14^+^ CD4^+^ monocytes (*r* = 0.68; *p <* 0.005) was found. This result could indicate that even if the expression of this co-receptor was not so different in both groups, stimulation with gp120 could induce an increase in TRAIL receptor expression, activation of axis TRAIL pathway and cell death independently of the entry receptor CD4, and this in turn could cause an augmented apoptosis sensibility in subjects which are going through a X4 tropism change mediated by the TRAIL pathway.

Immune activation is an essential topic in the pathogenesis of the disease due to it has been recognized that in HIV infection an activation state is related to a more efficient viral replication, an increase in the expression of more virulent strains, an impaired hematopoiesis, and apoptosis of CD4^+^ T-cells. Furthermore, a state of chronic activation favors the viral transmission, the progression of the disease, and in turn decreases the survival of patients [37]. The expression of CD80, CD86 and HLA-DR on CD14^+^ CD4^+^ monocytes from PLWHIV and HIV- controls was measured, without finding significant differences in the expression of any activation marker on these cells. According to our results, Stiksrud, et al. found no difference in HLA-DR expression between ART-*naïve* PLWHIV and the control group, however they reported an increase in the value of MFI of HLA-DR in total monocytes as well as in intermediate monocytes (CD14^++^ CD16^+^) from immunological non-responders (INRs) vs. Immunological Responders (IR) [38]. Again, the difference of their investigation with our study is the CD4^+^ T-cell count because they found more immune activation in PLWHIV with poor reconstitution (INR). This could contribute to the events typical of this group of PLWHIV, including, a high rate of apoptosis [39]. In vitro assays have reported an increase in the expression of CD80 and CD86 after exposure to HIV infection [40], Otherwise, in relation to apoptosis mediated by CD80 and CD86 expression, it is known that at least in B cells, CD80 and CD86 regulate the expression of pro-apoptotic as well as Fas and FasL expression and anti-apoptotic molecules; respectively. In macrophages the viability is improved after CD86 stimulation [41, 42]. Thus, the level of expression and function of CD80 and CD86, could have a greater impact in the progression of the disease in more advanced stages of the HIV infection. In relation to CD3^+^ CD4^+^ T-cells, we evaluated the expression of CD25, CD69 and CD38, finding only a significant increase in the expression of CD38 in cells from PLWHIV respect to HIV- controls. CD69 and CD25 have been classified as early activation markers on lymphocytes; CD69 is expressed 2–3 h after stimulation reaching its maximum levels within 12–24 h and is involved in the transmission of costimulatory signals, while the CD25 is part of the IL-2 receptor and is expressed around 24–48 h after mitogenic stimulation [43–45]. In relation to CD69 and in accordance with our results, no significant differences were reported between PLWHIV and healthy controls [43]. The presence of CD69 is related to insufficient activation signals, a decreased proliferation [46], death of monocytes [47] and apoptosis of activated T-cells [43, 48]. With respect to CD25 expression, the reports are contradictory; there are studies that, in agreement to our results, do not find a significant difference in the expression of CD25 on CD4^+^ T-cells; contradictory, Ramilo et al. through an in vitro assay showed a significant increase in CD25 in PLWHIV [49]. By maintaining the expression of CD25, the virus ensures a productive infection [34], however in HIV infection where is perpetuated a repeated stimulation of CD4^+^ T-cells, also is favored an activation induced cell death (AICD) result of the co-expression of Fas and FasL [50].

CD38 is an enzyme that hydrolyzes nicotinamide adenine dinucleotide (NAD^+^) to nicotinamide and ADP-ribose favoring the cellular metabolic rate in a state of activation [51]. CD38 is a marker of cellular activation [52, 53] and its elevated expression on CD8^+^ T-cells and in the context of HIV infection, is considered a predictor marker in the progression to AIDS and also it has been postulated as a marker of clinical management in PLWHIV [54]. The elevated expression of CD38 on CD4^+^ T-cells also is related to a poor prognosis in these subjects since it has been demonstrated that a decline important of CD4^+^ T-cells correspond to CD4^+^ CD38^+^ HLA-DR^+^ T-cell, moreover the loss of CD38^+^ CD4^+^ T-cells occurs in subjects with advanced infection [55–57]. On the other hand, the enzymatic activity of CD38 may protect lymphocytes from apoptosis, increasing the expression of molecules such as members of Bcl-2 family and NAm, this last inhibits HIV-1 replication at a post integrational level, and prevents apoptosis in T cells of PLWHIV [58].

Regarding the inflammatory cytokines, we did not observe a significant difference between IL1-β, IL-6, IL-8 and TNF-α, whereas in the case of IL-18 it was observed a significant elevation of the cytokine in sera of PLWHIV (*p <* 0.05, Fig. 8E). Diverse studies of cell cultures infected in vitro and monocytes cultures derived from patients, as well as measurements made on PLWHIV serum, which include untreated subjects, have reported elevated levels of cytokines such as: IL1-β, IL-6, IL-8 and TNF-α, while, levels that decrease after the initiation of ART, which might contribute to suppression of HIV replication and restoration of CD4^+^ T-cells. At this point, it is important to mention that the detection methods were different from the one used in this study [59, 60]. Recently, in previous study by our research group, it was reported that IL-1β and IL-8 are elevated in serum from INR ART-treated chronic PLWHIV compared to HIV- controls [61]. Otherwise, some investigations with the same interest groups as those in this study and, using a similar technique, did not report a significant difference in cytokine levels between PLWHIV before ART in an early and late infection; interestingly, and in accordance with our results, this study reports a significant increase of IL-18 in PLWHIV before ART, levels that are decreased, not at level of HIV- control, in subjects who starting their ART [62]. Thus, cytokine alterations vary over the course of HIV disease progression. In these sense it has been reported that in the acute phase of HIV infection, elevated plasma levels of inflammatory cytokines are associated with a high viral load and can estimate the time at which the CD4^+^ T-cells count will drop < 350 cells/µL; in this study, PLWHIV showed a CD4^+^ T-cells count > 400 cells/µL; this could explain that PLWHIV in this study still do not show a significant elevation of most of the evaluated cytokines [63]. The elevation of IL-18 in these PLWHIV could contribute to stimulate innate immunity and Th1 response by its ability to induce IFN-γ from T and natural killer (NK) cells. However, it has been hypothesized that chronic excessive production of IL-18 contributes to AIDS pathogenesis, since in vitro studies have shown that IL-18 improves HIV-1 replication in both monocytes and T-cells. In association with decreased levels of IL-12, the IL-18 promotes the differentiation of Th2 CD4^+^ T-cells in the later stages of the infection; in addition, it has been reported that IL-18 induce destruction of the central nervous system and other tissues, thus it has been suggested that elevated levels of IL-18 are associated with and may contribute to a virological treatment failure and disease progression in PLWHIV [30, 64]. It has been reported that, IL-18 boosts FasL expression on NK and CD8^+^ T-cells, additionally binding with Fas can induce IL-18 production [8]. In a study using PBMCs from PLWHIV, TRAIL expression was evaluated after IL-18 stimulation, in which an increase in ligand expression was observed as well as coreceptor CXCR4 [30]. On the other hand, in the present study we found a positive correlation with IL-18 levels and DR5 expression, both CD3^+^ CD4^+^ T-cells (*r* = 0.56; *p <* 0.05) and monocytes (*r* = 0.636; *p <* 0.05), (Fig. 9A and B; respectively). Thus, this receptor increase could be modulated by IL-18 secretion.

Finally, regarding the assays using the recombinant ligands and the Caspase 8 inhibitor. It was found that the Fas pathway behaves in a mostly homogeneous manner. In the CD3^+^ CD4^+^ T-cells, greater apoptosis was observed in PLWHIV. The functional activity of Fas pathway in this group could be explained by a c-FLIP overexpression, this molecule blocks the union of the pro-caspase 8 to the DISC and by that causes an apoptosis inhibition [65]. In the case of CD14^+^ CD4^+^ monocytes from HIV- controls the apoptosis decreased significantly when the ligand was added, a cellular response that was not observed in CD14^+^ CD3^+^ monocytes from PLWHIV. Instead, it was observed a slightly higher apoptosis, due to which it can be assume that cell death is carried out by a Fas independent mechanism.

In the case of the TRAIL pathway, and specifically in CD3^+^ CD4^+^ T-cells in early apoptosis a behavior very similar to Fas pathway was presented. However, it was observed that there was a significant increase in late apoptosis in CD3^+^ CD4^+^ T-cells and C14^+^ CD4^+^ monocytes from PLWHIV and HIV- controls, cellular event that in turn is decreased in a significant manner after adding the inhibitor. Therefore, it can be speculated that TRAIL pathway modulates the apoptosis in this population, and it is functional in PLWHIV contrary to what was observed on the Fas pathway. In the case of the CD14^+^ CD4^+^ monocytes from PLWHIV, there was an increase in early apoptosis in response to recombinant TRAIL ligand, an effect that was not inhibited by caspase 8 blockade. TRAIL activity independent to Caspase 8, has been reported in a mechanism that promotes necroptotic cell death by activation of RIPK [66], this could explain the lack of inhibition once we block caspase activity, adding to this, phosphatidyl serine externalization has been reported in initial stages of necroptosis [67] which explains why we can detect it by using Annexin V.

## Conclusions

Fas death pathway is considered one of the most important in the depletion of CD3^+^ CD4^+^ T-cells in primary HIV infection, in the present study Fas receptor and FasL expression have a discreate increase in CD3^+^ CD4^+^ T-cells whereas only FasL increase in CD14^+^ CD4^+^ monocytes from PLWHIV.

Regarding TRAIL receptors, the expression of DR5 was increased significantly in T -cells and monocytes and this expression could be related to HIV infection progression since, was positively associated with infection time. In addition, a positive correlation was observed between CXCR4 and DR5 in both CD14^+^ CD4^+^ monocytes and CD3^+^ CD4^+^ T-cells. Additionally, IL-18 was augmented in PLWHIV and correlated positively with the DR5 receptor and FasL in both cell types.

In the last 30 years the Fas pathway has been of significance regarding the depletion of CD4^+^ T lymphocytes in HIV infection, however in this study we got relevant findings in relation to the TRAIL pathway. We found a correlation between DR5 and disease progression which could signal this receptor as a biomarker for the progression of the disease, future studies focused on the role of DR5 could center their scope in the changes of DR5 in different stages of the disease. Additionally, a possible activation axis between IL-18 and DR5 would be of interest in a functional study in which stimulation of cells with the interleukin could modify expression and functionality of the TRAIL pathway in HIV infected cells and their bystanders.

In this study, Fas apoptotic functional assays showed no changes in both cells (under in vitro conditions). As regards TRAIL pathway, CD14^+^ CD4^+^ monocytes apoptosis was induced by TRAIL ligand under in vitro conditions, however only early apoptosis was detected; inhibition of TRAIL apoptotic pathway in monocytes was not achieved by the addition of Caspase-8 inhibitor.

Some of our results show a trend, and p-value is certainly very close to significance. This could be attributed to the sample size, so we consider that an increase, could improve the p-value and thereby achieve statistical significance. On the other hand, in HIV infection the number of cells is small, and this is a limitation when the objective is to evaluate a large number of variables. Additionally, an interesting perspective would be to be able to identify cells infected and uninfected with HIV, this could lead to some interesting results to look for in the future of this line of research. Further research, about the regulation of activation and inhibition of the TRAIL pathway in the context of HIV infection is needed.

## Data Availability

The datasets used and/or analyzed during the current study are available from the corresponding author on reasonable request.

## References

[1] Vijayan KV, Karthigeyan KP, Tripathi SP, Hanna LE. Pathophysiology of CD4 + T-Cell depletion in HIV-1 and HIV-2 infections. Front Immunol. 2017;8(MAY):257162. 10.3389/fimmu.2017.00580 PMC5440548 28588579

[2] Cummins NW, Badley AD. Making sense of how HIV kills infected CD4 T cells: implications for HIV cure. Mol Cell Ther. 2014;2:20. 10.1186/2052-8426-2-20 10.1186/2052-8426-2-20 PMC4452072 26056587

[3] Timilsina U, Gaur R. Modulation of apoptosis and viral latency– An axis to be well understood for successful cure of human immunodeficiency virus. J Gen Virol. 2016;97(4):813–24. 26764023 10.1099/jgv.0.000402

[4] Paim AC, Badley AD, Cummins NW. Mechanisms of human immunodeficiency Virus-Associated lymphocyte regulated cell death. AIDS Res Hum Retroviruses. 2020;36(2):101–15. 31659912 10.1089/aid.2019.0213 PMC7044792

[5] Cummins NW, Badley AD. Anti-apoptotic mechanisms of HIV: lessons and novel approaches to curing HIV. Cell Mol Life Sci. 2013;70(18):3355–63. 23275944 10.1007/s00018-012-1239-3 PMC3753464

[6] Yates A, Stark J, Klein N, Antia R, Callard R. Understanding the Slow Depletion of Memory CD4 + T Cells in HIV Infection. PLOS Med [Internet]. 2007 [cited 2023 Sep 14];4(5):e177. Available from: https://journals.plos.org/plosmedicine/article?id=10.1371/journal.pmed.0040177 10.1371/journal.pmed.0040177 PMC1872038 17518516

[7] Piconi S, Trabattoni D, Gori A, Parisotto S, Magni C, Meraviglia P et al. Immune activation, apoptosis, and treg activity are associated with persistently reduced CD4 + T-cell counts during antiretroviral therapy. AIDS [Internet]. 2010 Aug 24 [cited 2023 Sep 14];24(13):1991–2000. Available from: https://journals.lww.com/aidsonline/fulltext/2010/08240/immune_activation,_apoptosis,_and_treg_activity.3.aspx 10.1097/QAD.0b013e32833c93ce 20651586

[8] Kaplanski G. Interleukin-18: biological properties and role in disease pathogenesis. Immunol Rev. 2018;281(1):138–53. 29247988 10.1111/imr.12616 PMC7165732

[9] Kottilil S, Jackson JO, Reitano KN, O’Shea MA, Roby G, Lloyd M, et al. Innate immunity in HIV infection: enhanced susceptibility to CD95-mediated natural killer cell death and turnover induced by HIV viremia. J Acquir Immune Defic Syndr. 2007;46(2):151–9. 17558334 10.1097/QAI.0b013e3180dc9909

[10] Cummins NW, Badley AD. Mechanisms of HIV-associated lymphocyte apoptosis: 2010 [Internet]. Vol. 1, Cell Death and Disease. nature; 2010. Available from: 10.1038/cddis.2010.77 10.1038/cddis.2010.77 PMC3032328 21368875

[11] Dai X, Zhang J, Arfuso F, Chinnathambi A, Zayed ME, Alharbi SA et al. Targeting TNF-related apoptosis-inducing ligand (TRAIL) receptor by natural products as a potential therapeutic approach for cancer therapy. 10.1177/1535370215579167 [Internet]. 2015 Apr 7 [cited 2023 Sep 14];240(6):760–73. Available from: https://journals.sagepub.com/10.1177/1535370215579167 10.1177/1535370215579167 PMC4935211 25854879

[12] Herbeuval J-P, Grivel J-C, Boasso A, Hardy AW, Chougnet C, Dolan MJ, et al. CD4 T-cell death induced by infectious and noninfectious HIV-1: role of type 1 interferon-dependent, TRAIL/DR5-mediated apoptosis. Blood. 2005;106(10):3524–31. 10.1182/blood-2005-03-1243 10.1182/blood-2005-03-1243 PMC1895067 16046522

[13] Herbeuval J, Grivel J, Boasso A, Hardy AW, Chougnet C, et al. CD4 ϩ T-cell death induced by infectious and noninfectious HIV-1: role of type 1 interferon– dependent, TRAIL / DR5-mediated apoptosis. Blood. 2005;106(10):3524–31. 10.1182/blood-2005-03-1243 10.1182/blood-2005-03-1243 PMC1895067 16046522

[14] Miura Y, Misawa N, Maeda N, Inagaki Y, Tanaka Y, Ito M et al. Critical Contribution of Tumor Necrosis Factor–Related Apoptosis-Inducing Ligand (Trail) to Apoptosis of Human Cd4 + T Cells in HIV-1–Infected Hu-Pbl-Nod-Scid Mice. J Exp Med [Internet]. 2001 Mar 5 [cited 2023 Sep 14];193(5):651–60. Available from: http://www.jem.org/cgi/content/full/193/5/651 10.1084/jem.193.5.651 PMC2193390 11238596

[15] Lum JJ, Pilon AA, Sanchez-Dardon J, Phenix BN, Kim JE, Mihowich J et al. Induction of Cell Death in Human Immunodeficiency Virus-Infected Macrophages and Resting Memory CD4 T Cells by TRAIL/Apo2L. J Virol [Internet]. 2001;75(22):11128–36. Available from: http://jvi.asm.org/cgi/content/abstract/75/22/11128 10.1128/JVI.75.22.11128-11136.2001 PMC114692 11602752

[16] Van Grevenynghe J, Cubas RA, Noto A, DaFonseca S, He Z, Peretz Y et al. Loss of memory B cells during chronic HIV infection is driven by Foxo3a- and TRAIL-mediated apoptosis. J Clin Invest [Internet]. 2011 Oct 3 [cited 2023 Sep 14];121(10):3877–88. Available from: http://www.jci.org 10.1172/JCI59211 PMC3195482 21926463

[17] Yang Y, Tikhonov I, Ruckwardt TJ, Zapata JC, Pauza CD, Maria S, et al. Monocytes treated with human immunodeficiency virus tat kill uninfected CD4 + Cells by a Tumor necrosis ligand-mediated mechanism monocytes treated with human immunodeficiency virus tat kill uninfected CD4 ϩ cells by a tumor necrosis factor-related apopto. J Virol. 2003. 10.1128/jvi.77.12.6700-6708.2003 10.1128/JVI.77.12.6700-6708.2003 PMC156176 12767990

[18] Zhu DM, Shi J, Liu S, Liu Y, Zheng D. HIV infection enhances TRAIL-induced cell death in macrophage by down-regulating decoy receptor expression and generation of reactive oxygen species. PLoS ONE. 2011;6(4):1–9. 10.1371/journal.pone.0018291 PMC3071698 21483669

[19] Barblu L, Smith N, Durand S, Scott-Algara D, Boufassa F, Delfraissy JF et al. Reduction of death receptor 5 expression and apoptosis of CD4 + T cells from HIV controllers. Clin Immunol [Internet]. 2014;155(1):17–26. Available from: 10.1016/j.clim.2014.07.010 10.1016/j.clim.2014.07.010 25110157

[20] Sloand EM, Young NS, Kumar P, Weichold FF, Sato T, Maciejewski JP. Role of Fas ligand and receptor in the mechanism of T-cell depletion in acquired immunodeficiency syndrome: Effect on CD4 + lymphocyte depletion and human immunodeficiency virus replication. Blood [Internet]. 1997 Feb 15 [cited 2019 Nov 17];89(4):1357–63. Available from: https://ashpublications.org/blood/article/89/4/1357/139075/Role-of-Fas-Ligand-and-Receptor-in-the-Mechanism 9028959

[21] Hosaka N, Oyaizu N, Ikehara S, Pahwa S. Expressions of Fas (Cd95) and Fas Ligand in HIV Infected Individuals. Mol Biol Hematop 6 [Internet]. 1999 [cited 2023 Sep 20];257–61. Available from: https://link.springer.com/chapter/10.1007/978-1-4615-4797-6_32

[22] Mitra D. HIV-1 upregulates Fas ligand expression in CD4 + T cells in vitro and in vivo: Association with Fas-mediated apoptosis and modulation by aurintricarboxylic acid [Internet]. Vol. 87, Immunology. 1996 [cited 2019 May 22]. Available from: https://onlinelibrary.wiley.com/doi/pdf/10.1046/j.1365-2567.1996.510589.x 10.1046/j.1365-2567.1996.510589.x PMC1384136 8675212

[23] Blanco R, Carrasco L, Ventoso I. Cell killing by HIV-1 protease. J Biol Chem [Internet]. 2003 Jan 10 [cited 2019 May 19];278(2):1086–93. Available from: http://www.ncbi.nlm.nih.gov/pubmed/12370191 10.1074/jbc.M205636200 12370191

[24] Cummins NW, Badley AD. Mechanisms of HIV-associated lymphocyte apoptosis: 2010 [Internet]. Cell Death and Disease nature; 2010. Available from: 10.1038/cddis.2010.77 10.1038/cddis.2010.77 PMC3032328 21368875

[25] Jeremias I, Herr I, Boehler T, Debatin KM. TRAIL/Apo-2-ligand-induced apoptosis in human T cells. Eur J Immunol [Internet]. 1998 Jan 1 [cited 2018 Nov 14];28(1):143–52. Available from: http://doi.wiley.com/10.1002/%28SICI%291521-4141%28199801%2928%3A01%3C143%3A%3AAID-IMMU143%3E3.0.CO%3B2-3 10.1002/(SICI)1521-4141(199801)28:01<143::AID-IMMU143>3.0.CO;2-3 9485194

[26] Somma F, Tuosto L, Gilardini Montani MS, Di Somma MM, Cundari E, Piccolella E. Engagement of CD4 before TCR triggering regulates both Bax- and Fas (CD95)-mediated apoptosis. J Immunol [Internet]. 2000 May 15 [cited 2019 May 19];164(10):5078–87. Available from: http://www.ncbi.nlm.nih.gov/pubmed/9531275 10.4049/jimmunol.164.10.5078 10799864

[27] Estaquier J, Tanaka M, Suda T, Nagata S, Golstein P, Ameisen JC. Fas-mediated apoptosis of CD4 + and CD8 + T cells from human immunodeficiency virus-infected persons: differential in vitro preventive effect of cytokines and protease antagonists. Blood. 1996;87(12):4959–66. 8652808

[28] Shepard BD, De Forni D, McNamara DR, Foli A, Rizza SA, Abraham RS, et al. Beneficial effect of TRAIL on HIV burden, without detectable immune consequences. PLoS ONE. 2008;3(8):e3096. 18769477 10.1371/journal.pone.0003096 PMC2517653

[29] Herbeuval JP, Boasso A, Grivel JC, Hardy AW, Anderson SA, Dolan MJ, et al. TNF-related apoptosis-inducing ligand (TRAIL) in HIV-1-infected patients and its in vitro production by antigen-presenting cells. Blood. 2005;105(6):2458–64. 15585654 10.1182/blood-2004-08-3058

[30] Stylianou E, Bjerkeli V, Yndestad A, Heggelund L, Wæhre T, Damås JK, et al. Raised serum levels of interleukin-18 is associated with disease progression and May contribute to virological treatment failure in HIV-1-infected patients. Clin Exp Immunol. 2003;132(3):462–6. 12780693 10.1046/j.1365-2249.2003.02179.x PMC1808719

[31] Nie Z, Aboulnasr F, Natesampillai S, Burke SP, Krogman A, Bren GD et al. Both HIV-Infected and Uninfected Cells Express TRAILshort, Which Confers TRAIL Resistance upon Bystander Cells within the Microenvironment. J Immunol [Internet]. 2017;ji1701113. Available from: http://www.jimmunol.org/lookup/doi/10.4049/jimmunol.1701113 10.4049/jimmunol.1701113 PMC5808399 29263214

[32] Herbeuval J-P, Nilsson J, Boasso A, Hardy AW, Kruhlak MJ, Anderson SA, et al. Differential expression of IFN- and TRAIL/DR5 in lymphoid tissue of progressor versus nonprogressor HIV-1-infected patients. Proc Natl Acad Sci. 2006;103(18):7000–5. 16632604 10.1073/pnas.0600363103 PMC1444883

[33] Clapham PR, McKnight Á. HIV-1 receptors and cell tropism. Br Med Bull. 2001;58:43–59. 11714623 10.1093/bmb/58.1.43

[34] Lum JJ, Schnepple DJ, Badley AD. Acquired T-cell sensitivity to TRAIL mediated killing during HIV infection is regulated by CXCR4-gp120 interactions. Aids. 2005;19(11):1125–33. 15990565 10.1097/01.aids.0000176212.16205.23

[35] Biard-Piechaczyk M, Robert-Hebmann V, Richard V, Roland J, Hipskind RA, Devaux C. Caspase-dependent apoptosis of cells expressing the chemokine receptor CXCR4 is induced by cell membrane-associated human immunodeficiency virus type 1 envelope glycoprotein (gp120). Virology [Internet]. 2000 [cited 2019 Nov 20];268(2):329–44. Available from: http://www.idealibrary.com 10.1006/viro.1999.0151 10704341

[36] Alkhatib G. The biology of CCR5 and CXCR4. Curr Opin HIV AIDS. 2009. https://journals.lww.com/co-hivandaids/abstract/2009/03000/the_biology_of_ccr5_and_cxcr4.5.aspx 10.1097/COH.0b013e328324bbec PMC2718543 19339947

[37] Stiksrud B, Aass HCD, Lorvik KB, Ueland T, Trøseid M, Dyrhol-Riise AM. Activated dendritic cells and monocytes in HIV immunological nonresponders: HIV-induced interferon-inducible protein-10 correlates with low future CD4 + recovery. AIDS [Internet]. 2019 Jun 1 [cited 2023 Sep 20];33(7):1117–29. Available from: https://journals.lww.com/aidsonline/fulltext/2019/06010/activated_dendritic_cells_and_monocytes_in_hiv.2.aspx 10.1097/QAD.0000000000002173 PMC6511429 30789356

[38] Bertho N, Drénou B, Laupeze B, Berre C, Le, Amiot L, Grosset J-M et al. HLA-DR-Mediated Apoptosis Susceptibility Discriminates Differentiation Stages of Dendritic/Monocytic APC. J Immunol [Internet]. 2000 Mar 1 [cited 2023 Sep 20];164(5):2379–85. Available from: 10.4049/jimmunol.164.5.2379 10.4049/jimmunol.164.5.2379 10679073

[39] Gaardbo JC, Hartling HJ, Gerstoft J, Nielsen SD. Incomplete immune recovery in HIV infection: Mechanisms, relevance for clinical care, and possible solutions. Clin Dev Immunol. 2012;2012. 10.1155/2012/670957 PMC3312328 22474480

[40] Wang X, Lewis DE. CD86 expression correlates with amounts of HIV produced by macrophages in vitro. J Leukoc Biol [Internet]. 2001 Mar 1 [cited 2023 Sep 20];69(3):405–13. Available from: https://onlinelibrary.wiley.com/doi/full/10.1189/jlb.69.3.405 11261787

[41] Testi R, Phillips JH, Lanier LL. Baltimore, Md. T cell activation via Leu-23 (CD69). J Immunol (1950). 1989;143(4):1123–8. 2501389

[42] Nielsen SD, Afzelius P, Ersbøll AK, Nielsen JO, Hansen JES. Expression of the activation antigen CD69 predicts functionality of in vitro expanded peripheral blood mononuclear cells (PBMC) from healthy donors and HIV-infected patients. Clin Exp Immunol. 1998;114(1):66–72. 9764605 10.1046/j.1365-2249.1998.00685.x PMC1905088

[43] Sieg SF, Harding CV, Lederman MM. HIV-1 infection impairs cell cycle progression of CD4 + T cells without affecting early activation responses. J Clin Invest. 2001;108(5):757–64. 11544282 10.1172/JCI12685 PMC209381

[44] Pitsios C, Dimitrakopoulou A, Tsalimalma K, Kordossis T, Choremi-Papadopoulou H. Expression of CD69 on T-cell subsets in HIV‐1 disease. Scand J Clin Lab Invest [Internet]. 2008 May 1 [cited 2023 Sep 20];68(3):233–41. Available from: https://www.tandfonline.com/doi/abs/10.1080/00365510701630227 10.1080/00365510701630227 17917998

[45] Böhler T, Walcher J, Hölzl-Wenig G, Schnitzler P, Geiss M, Buchholz B et al. Expression of CD69 on T-cells from HIV-1-infected children and adolescents increases with increasing viral load. Eur J Pediatr [Internet]. 1999 [cited 2023 Sep 20];158(8):638–44. Available from: https://link.springer.com/article/10.1007/s004310051167 10.1007/s004310051167 10445342

[46] Ramírez R, Carracedo J, Castedo M, Zamzami N, Kroemer G. CD69-Induced monocyte apoptosis involves multiple nonredundant signaling pathways. Cell Immunol. 1996;172(2):192–9. 8964080 10.1006/cimm.1996.0232

[47] Esplugues E, Sancho D, Vega-Ramos J, Martínez-A C, Syrbe U, Hamann A et al. Enhanced Antitumor Immunity in Mice Deficient in CD69. J Exp Med [Internet]. 2003 May 5 [cited 2023 Sep 20];197(9):1093–106. Available from: http://www.jem.org/cgi/doi/10.1084/jem.20021337 10.1084/jem.20021337 PMC2193974 12732655

[48] Zola H, Koh LY, Mantzioris BX, Rhodes D. Patients with HIV infection have a reduced proportion of lymphocytes expressing the IL2 receptor p55 chain (TAC, CD25). Clin Immunol Immunopathol. 1991;59(1):16–25. 1708315 10.1016/0090-1229(91)90078-o

[49] Ramilo O, Bell KD, Uhr JW, Vitetta ES. Role of CD25 + and CD25-T cells in acute HIV infection in vitro. J Immunol [Internet]. 1993 Jun 1 [cited 2023 Sep 20];150(11):5202–8. Available from: 10.4049/jimmunol.150.11.5202 8496611

[50] Malavasi F, Funaro A, Roggero S, Horenstein A, Calosso L, Mehta K. Human CD38: a glycoprotein in search of a function. Immunol Today. 1994;15(3):95–7. 8172650 10.1016/0167-5699(94)90148-1

[51] Funaro A, Spagnoli GC, Ausiello CM, Alessio M, Roggero S, Delia D et al. Involvement of the multilineage CD38 molecule in a unique pathway of cell activation and proliferation. J Immunol [Internet]. 1990 Oct 15 [cited 2023 Sep 20];145(8):2390–6. Available from: 10.4049/jimmunol.145.8.2390 1976692

[52] Liu Z, Cumberland WG, Hultin LE, Prince HE, Detels R, Giorgi JV. Elevated CD38 antigen expression on CD8 + T cells is a stronger marker for the risk of chronic HIV disease progression to AIDS and death in the multicenter AIDS cohort study than CD4 + cell count, soluble immune activation markers, or combinations of HLA-DR. JAIDS J Acquir Immune Defic Syndr. 1997;16(2):83–92. 10.1097/00042560-199710010-00003 9358102

[53] Liu Z, Hultin LE, Cumberland WG, Hultin P, Schmid I, Matud JL, et al. Elevated relative fluorescence intensity of CD38 antigen expression on CD8 + T cells is a marker of poor prognosis in HIV infection: results of 6 years of follow up. Commun Clin Cytom. 1996. https://europepmc.org/article/med/8809474 10.1002/(SICI)1097-0320(19960315)26:1<1::AID-CYTO1>3.0.CO;2-L 8809474

[54] Benito JM, Zabay JM, Gil J, Bermejo M, Escudero A, Sánchez E, et al. Quantitative alterations of the functionally distinct subsets of CD4 and CD8 T lymphocytes in asymptomatic HIV infection: changes in the expression of CD45RO, CD45RA, CD11b, CD38, HLA-DR, and CD25 antigens. JAIDS J Acquir Immune Defic Syndr. 1997;14(2):128–35. 10.1097/00042560-199702010-00005 9052721

[55] Giorgi JV, Hultin LE, McKeating JA, Johnson TD, Owens B, Jacobson LP et al. Shorter Survival in Advanced Human Immunodeficiency Virus Type 1 Infection Is More Closely Associated with T Lymphocyte Activation than with Plasma Virus Burden or Virus Chemokine Coreceptor Usage. J Infect Dis [Internet]. 1999 Apr 1 [cited 2023 Sep 20];179(4):859–70. Available from: 10.1086/314660 10.1086/314660 10068581

[56] Rodríguez-Alba JC, Abrego-Peredo A, Gallardo-Hernández C, Pérez-Lara J, Santiago-Cruz W, Jiang W et al. HIV Disease Progression: Overexpression of the Ectoenzyme CD38 as a Contributory Factor? BioEssays [Internet]. 2019 Jan 1 [cited 2023 Sep 20];41(1):1800128. Available from: https://onlinelibrary.wiley.com/doi/full/10.1002/bies.201800128 10.1002/bies.201800128 PMC6545924 30537007

[57] Sandoval-Montes C, Santos-Argumedo L. CD38 is expressed selectively during the activation of a subset of mature T cells with reduced proliferation but improved potential to produce cytokines. J Leukoc Biol [Internet]. 2005 Jan 3 [cited 2023 Sep 20];77(4):513–21. Available from: 10.1189/jlb.0404262 10.1189/jlb.0404262 15618297

[58] Savarino A, Bottarel F, Malavasi F, Dianzani U. Role of CD38 in HIV-1 infection: an epiphenomenon of T-cell activation or an active player in virus/host interactions? Aids. 2000;14(9):1079–89. 10894271 10.1097/00002030-200006160-00004

[59] Kedzierska K, Crowe SM. Review Cytokines and HIV-1: interactions and clinical implications. Antivir Chem Chemother [Internet]. 2001;12(3):133–50. Available from: http://journals.sagepub.com/doi/pdf/10.1177/095632020101200301 10.1177/095632020101200301 12959322

[60] Osuji FN, Onyenekwe CC, Ahaneku JE, Ukibe NR. The effects of highly active antiretroviral therapy on the serum levels of pro-inflammatory and anti-inflammatory cytokines in HIV infected subjects. J Biomed Sci [Internet]. 2018 Dec 3 [cited 2023 Sep 20];25(1):1–8. Available from: https://jbiomedsci.biomedcentral.com/articles/10.1186/s12929-018-0490-9 10.1186/s12929-018-0490-9 PMC6276218 30501642

[61] Ruiz-Briseño MR, De Arcos-Jiménez JC, Ratkovich-González S, Sánchez-Reyes K, González-Hernández LA, Andrade-Villanueva JF et al. Association of intestinal and systemic inflammatory biomarkers with immune reconstitution in HIV + patients on ART. J Inflamm (United Kingdom). 2020;17(1). 10.1186/s12950-020-00262-4 PMC7558748 33071649

[62] Vanpouille C, Introini A, Morris SR, Margolis L, Daar ES, Dube MP et al. Distinct Cytokine/chemokine Network in Semen and Blood Characterize Different Stages of HIV Infection HHS Public Access. AIDS [Internet]. 2016 [cited 2018 Nov 15];30(2):193–201. Available from: http://links.lww.com/QAD/A830 10.1097/QAD.0000000000000964 PMC4862605 26558730

[63] Roberts L, Passmore JAS, Williamson C, Little F, Bebell LM, Mlisana K, et al. Plasma cytokine levels during acute HIV-1 infection predict HIV disease progression. AIDS. 2010. 10.1097/QAD.0b013e3283367836 10.1097/QAD.0b013e3283367836 PMC3001189 20224308

[64] Ahmad R, Sindhu STA, Toma E, Morisset R, Ahmad A. Elevated levels of Circulating interleukin-18 in human immunodeficiency virus-infected individuals: role of peripheral blood mononuclear cells and implications for AIDS pathogenesis. J Virol. 2002;76(24):12448–56. 12438570 10.1128/JVI.76.24.12448-12456.2002 PMC136707

[65] Irmler M, Thome M, Hahne M, Schneider P, Hofmann K, Steiner V et al. Inhibition of death receptor signals by cellular FLIP. Nature [Internet]. 1997 Jul [cited 2019 May 15];388(6638):190–5. Available from: http://www.nature.com/articles/40657 10.1038/40657 9217161

[66] Jouan-Lanhouet S, Arshad MI, Piquet-Pellorce C, Martin-Chouly C, Le Moigne-Muller G, Van Herreweghe F et al. TRAIL induces necroptosis involving RIPK1/RIPK3-dependent PARP-1 activation. Cell Death Differ 2012 1912 [Internet]. 2012 Jul 20 [cited 2022 Oct 11];19(12):2003–14. Available from: https://www.nature.com/articles/cdd201290 10.1038/cdd.2012.90 PMC3504714 22814620

[67] Zargarian S, Shlomovitz I, Erlich Z, Hourizadeh A, Ofir-Birin Y, Croker BA et al. Phosphatidylserine externalization, necroptotic bodies release, and phagocytosis during necroptosis. PLOS Biol [Internet]. 2017 Jun 26 [cited 2022 Oct 11];15(6):e2002711. Available from: https://journals.plos.org/plosbiology/article?id=10.1371/journal.pbio.2002711 10.1371/journal.pbio.2002711 PMC5501695 28650960

